# Goniodomic Acid, a Transient Oxirane Intermediate in the Conversion of the Macrolide Algal Toxin Goniodomin A to Seco Acids

**DOI:** 10.1021/acs.chemrestox.4c00390

**Published:** 2024-12-23

**Authors:** Constance M. Harris, Bernd Krock, Thomas M. Harris

**Affiliations:** † Department of Chemistry, Vanderbilt University, Nashville, Tennessee 37235, United States; ‡ Alfred-Wegener-Institut Helmholtz-Zentrum für Polar- und Meeresforschung (AWI), 27570 Bremerhaven, Germany

## Abstract

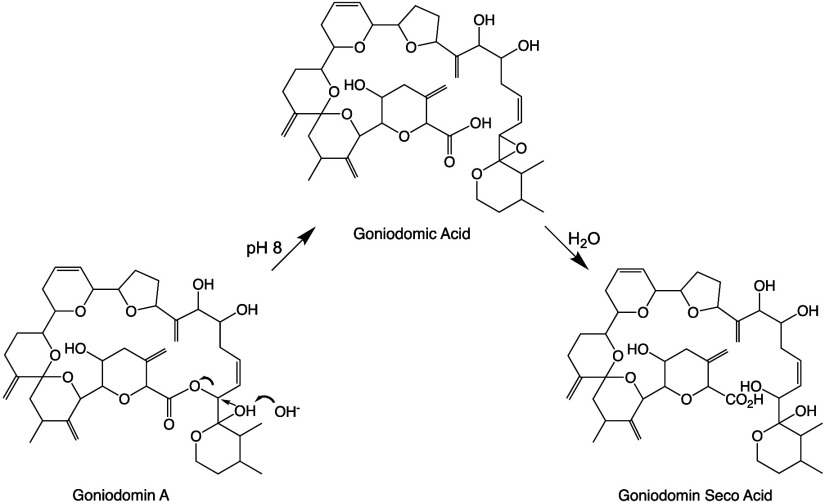

The algal macrolide goniodomin A (GDA) undergoes ring-cleavage under unusually mild, alkaline conditions to form mixtures of stereoisomers of seco acids GDA-sa and iso-GDA-sa. In the primary fragmentation pathway, opening of the macrolide ring occurs by displacement of the carboxyl group by a base-catalyzed attack of the C32 hemiketal hydroxy group on C31, yielding an oxirane-carboxylic acid, named goniodomic acid. The oxirane ring is unstable, undergoing solvolytic opening to form mainly GDA-sa. Experimental support for this pathway obtained by carrying out the ring-opening reaction in H_2_^18^O resulted in incorporation of the isotopic label at C32 of the seco acid. Collision-induced dissociation (CID) mass spectrometry of Na^+^ and NH_4_^+^ ion adducts was employed to establish that ring-opening of the macrolide ring occurred by alkyl-O cleavage. Fragmentation was dominated by Grob–Wharton decarboxylation and retro-Diels–Alder reactions of the labeled seco acids. Direct observation of goniodomic acid was achieved when the ring-opening reaction was carried out under anhydrous conditions. A minor alkyl-O cleavage pathway gives rise to iso-GDA-sa by allylic attack at C29 of GDA or of the oxirane. In the formation of both GDA-sa and iso-GDA-sa, ring-opening is likely to be catalyzed by Na^+^ and NH_4_^+^. Reversal of GDA-sa formation can occur in the mass spectrometer. CID fragmentation of the ^18^O-labeled GDA-sa restores the oxirane ring but causes preferential loss of the ^18^O label from C32.

The *Alexandrium* genus of dinoflagellates contains numerous members and is widespread throughout the marine world.^1^ Those that produce saxitoxins have been studied extensively due to these compounds being neurotoxins responsible for paralytic shellfish poisoning in humans. Lesser known are those that produce goniodomins (GDs). Six GD-producing species are known: *Alexandrium taylorii*, *Alexandrium hiranoi*, *Alexandrium pseudogonyaulax*, *Alexandrium limii*, *Alexandrium ogatae*, and *Alexandrium monilatum*.^2−6^ The GDs are polyketide macrolides with complex structures. The structure of the principal GD, goniodomin A (GDA, **1**), shown in Figure 1, has been established by NMR spectroscopy and X-ray.^4,7,8^

**Figure 1:**
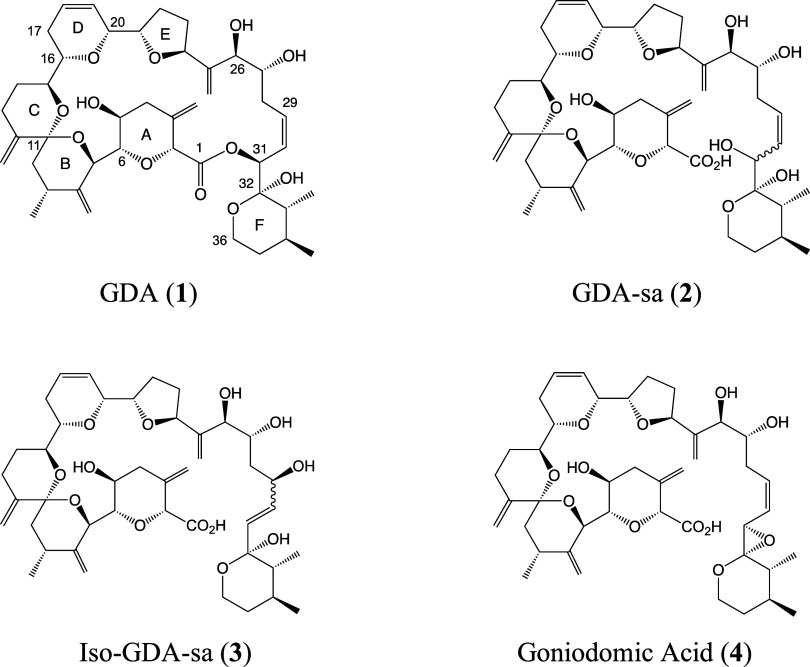
Structures of goniodomins **1**–**4**. Stereochemistry of C29–C31 of **2** and **3** not fully defined.

Toxicological studies of GDA reveal a mode of action involving interaction with actin.^9−13^ GDA is structurally similar to the pectenotoxins (e.g., PTX-2), which are macrolides produced by another dinoflagellate genus *Dinophysis*. They also interact with actin.^14^ One notable difference between the chemistry of GDA and that of PTXs is that the latter are more resistant to hydrolysis of the lactone moiety, whereas GDA readily undergoes ring-opening to form seco acids **2** and **3** and related structures under mild conditions, for example, by treatment with pH 8 seawater at ambient temperatures.^15^ The conversion of PTX-2 to the seco acid is regarded as being a detoxification process. Certain shellfish have been found to produce esterases that convert pectenotoxins to seco acids.^16^ Formation of the seco acids of GDA-sa may also be a detoxification process but the fact that the ring-opening process is spontaneous suggests that the seco acids may play an ecological role for the *Alexandrium* species that produce them. Studies of the toxicological properties of GDA and its transformation products are ongoing.

Structural characterization of GDA-sa by NMR and X-ray has been precluded by isomerization creating dynamic mixtures of stereoisomers. We recently reported characterization of this mixture by resorting to mass spectrometry as the structural tool.^15^ Working at pH 8.0 in 1:1 MeOH-H_2_O, methanolysis was shown to occur mainly by alkyl-O cleavage. Those studies did not address the pathway of hydrolysis but the hypothesis was put forward that hydrolytic ring-opening of GDA also involves alkyl-O cleavage of the lactone rather than the acyl-O cleavage observed with most lactones and other esters.

Hess and Smentek, employing density function theory (DFT), concluded that the facile ring-cleavage of GDA occurred by internal displacement with attack of the C32 hemiketal hydroxy group on C31 to form an oxirane ring (**4**) by displacement of the carboxy group as shown in Scheme 1.^17^ The oxirane ring then underwent ring-opening to form seco acids **2**.

**Scheme 1:**
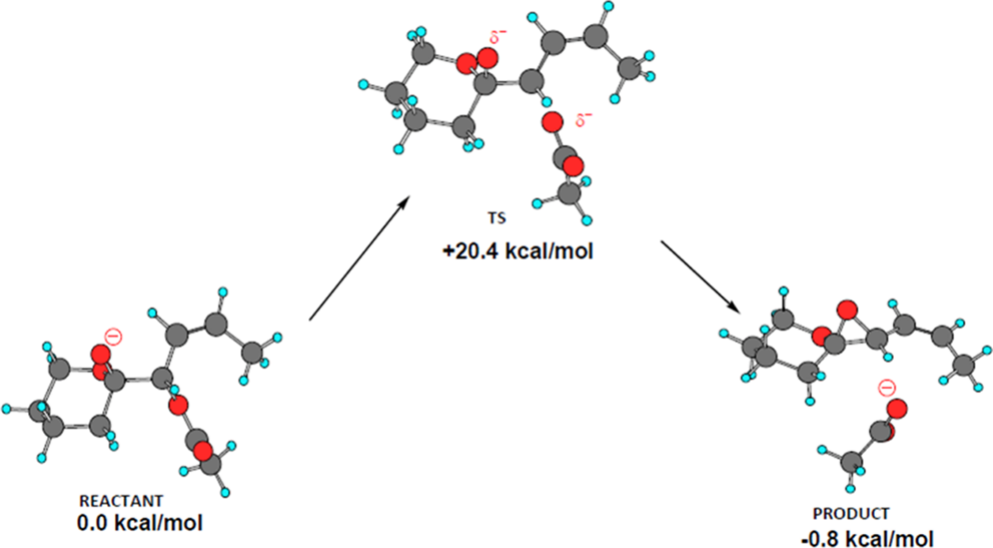
Intramolecular S_N_2 Reaction of GDA at pH 8 Involving Removal of the Proton from C32-OH and the Resulting Hydroxy Anion Displacing the Carboxy Group from C31 to Yield the Oxirane Ring of **4**(17) Used with permission of the journal.

We were skeptical of their oxirane proposal because our studies had failed to produce evidence for an oxirane intermediate. We concluded that a more likely ring-opening process involved allylic attack at C29 to give stereoisomers of iso-GDA-sa (**3**). Further investigation of the process by which ring-opening occurred has now provided us with new insight as to the mechanism of this unusual process of ring cleavage. Studies under anhydrous conditions at higher pH have made it possible to observe the formation of the oxirane intermediate and to follow subsequent ring-opening of the oxirane to form seco acids **2** and **3**.

## 2 Material and Methods

### 2.1 Materials

GDA was isolated by a previously described procedure from *A. monilatum* cells that had been collected via plankton nets from natural blooms in the York River, VA.^18^ MeOH and other solvents used for reactions were ACS grade. High-performance liquid chromatography (HPLC) analyses and separations were carried out with chromatography grade reagents. Reagents for MS and liquid chromatography–mass spectrometry (LC-MS) analyses were mass spectrometry grade. Milli-Q deionized water was employed for reactions and HPLC grade water was used for chromatography.

### 2.2 Reactions of GDA with pH 8.0 Sodium Phosphate Buffer in ^16^O and ^18^O 1:1 MeOH/H_2_O

The reaction of GDA with pH 8.0 buffer was carried out by combining 25 μg of GDA dissolved in 250 μL of MeOH with 250 μL of 100 μM sodium phosphate buffer (pH 8.0) in H_2_^16^O to create a homogeneous mixture, following guidelines from Harris et al.^18^ The reaction was allowed to proceed for 5 days at 30 °C at which point HPLC analysis indicated that the GDA had been expended. The sample was evaporated to dryness *in vacuo* (Savant SpeedVac) and the residue was taken up in 3 × 1.0 mL of C_6_H_6_ and centrifuged to precipitate sodium phosphate. Experimentation had demonstrated that the Na^+^ salt of GDA-sa was soluble in C_6_H_6_ at this concentration. With each trituration, supernatant was withdrawn with care being taken to avoid transfer of sodium phosphate. The combined supernatants were divided equally between two HPLC vials, evaporated to dryness and then taken up in MeOH for analysis by FT-ICR and UPLC-TQ mass spectrometry. The same procedures were used for the reaction in H_2_^18^O with the exception that the 250 μL solution of 100 μM sodium phosphate buffer (pH 8.0) was evaporated to dryness (Savant SpeedVac) and the H_2_O replaced with 250 μL of H_2_^18^O.

### 2.3 Reaction of GDA with Na_2_CO_3_ and NH_3_ in Anhydrous MeOH

The Na_2_CO_3_ reaction was carried out by adding 1.5 mL of an anhydrous, methanolic solution of Na_2_CO_3_ (7 mM) to 100 μg of GDA. The solution was stored for 4 days at ambient temperature, at which time HPLC indicated that GDA was depleted. The solution was evaporated to dryness *in vacuo* (SpeedVac). The residue was partitioned between H_2_O and CH_2_Cl_2_ with centrifugation to separate the layers. The aqueous layer was discarded. The organic layer was evaporated *in vacuo* to leave a white powder that was used for FT-ICR MS analysis after being taken up in MeOH.

Reactions of GDA with methanolic NH_3_ were carried out using 1.5 mL of 20 and 200 mM NH_3_ for 5 days at ambient temperature. The mixtures were evaporated to dryness with SpeedVac and the residues taken up in MeOH for C18 HPLC with a H_2_O-ACN gradient. The 20 mM reaction showed low yields of products and was abandoned. The 200 mM reaction was near completion. Two peaks were observed, a large polar one eluting immediately after the void volume and a small one at ∼8 min. A small-scale preparative separation was carried out to prepare samples for mass spectrometric analysis.

### 2.4 High-Resolution Mass Spectrometry

High-resolution mass spectra were acquired with a Bruker 10 T APEX-Qe FT-ICR mass spectrometer (Old Dominion Univ., Norfolk, VA, USA) using positive ion electrospray ionization. In all cases, the samples were introduced by direct infusion of a MeOH solution using a syringe pump because the instrument was not interfaced with an HPLC. Na^+^ adducts were observed using adventitious Na^+^ contained in the samples. Collision-induced dissociation (CID) spectra were acquired using argon as the collision gas. A 15 Da isolation window was employed with the CID voltage optimized at −39.3 V. Empirical formulas were assigned using the ChemCalc program.^19^

### 2.5 Tandem Mass Spectrometry

Samples were analyzed by ultrahigh-performance liquid chromatography (UPLC) coupled with tandem quadrupole mass spectrometry. The UPLC system consisted of a column oven, an autosampler and a binary pump (ACQUITY I UPLC Class, Waters, Eschborn, Germany) and was coupled to a triple quadrupole mass spectrometer (Xevo TQ-XS, Waters). The autosampler was held at 10 °C and sample separation was performed on a RP-18 column (PurospherSTAR end-capped (2 μm) Hibar HR 50–2.1, Merck, Darmstadt, Germany) equipped with a precolumn (0.5 μm, OPTI-SOLV EXP, Sigma-Aldrich, Hamburg, Germany) held at 40 °C. An alkaline elution system was used for NH_4_^+^ adducts with eluent A consisting of 6.7 mM aqueous NH_3_ and eluent B 9:1 (v/v) ACN and 6.7 mM aqueous NH_3_. For measurements of Na^+^ adducts an acidic system was used with eluent A consisting of 0.2% formic acid and 0.004% aqueous NH_3_ and eluent B of 0.2% formic acid and 0.004% aqueous NH_3_ in ACN. The flow rate was 0.6 mL min^–1^ and initial conditions of 5% B were held for 1.5 min. A linear gradient from 5% B to 100% B was performed within 2 min (until 3.5 min) followed by isocratic elution with 100% B for 3 min (until 6.5 min) prior to returning to initial conditions within 0.5 and 1 min equilibration time (total run time: 8 min).

Dwell times, cone voltage and collision energy used in selected reaction monitoring (SRM) experiments in the positive ionization mode were 0.06 s, 40 V, and 40 eV, respectively. The applied mass transitions are listed in Table 1 and the mass spectrometric parameters are given in Table 2. The collision energies for NH_4_^+^ adducts were 30 eV and for Na^+^ adducts 45 eV. Data were acquired and analyzed with MassLynx (Version 4.2, Waters).

**Table 1:** Compound Names, Screened Adducts, and Mass Transitions for Observing Fragmentation of ^16^O-GDA-sa and ^18^O-GDA-sa

compound	adduct	transition
^16^O-GDA-sa	NH_4_^+^	804.5 > 139.5
^16^O-GDA-sa	Na^+^	809.5 > 765.5
^18^O-GDA-sa	NH_4_^+^	806.5 > 139.5
^18^O-GDA-sa	Na^+^	811.5 > 767.5

**Table 2:** Mass Spectrometric Parameters of CID Experiments

parameter	setting
capillary voltage	3 kV
source temperature	150 °C
desolvation temperature	600 °C
desolvation gas	N_2_, 1000 L h^–1^
cone gas	150 L h^–1^
cone voltage	40 V
nebulizer gas	7.0 bar
collision gas flow	0.15 mL min^–1^
scan time	0.072 s

## 3 Results

### 3.1 Formation of ^16^O and ^18^O-Labeled GDA-sa

The hydrolysis of GDA gave GDA-sa (**2**) plus small amounts of iso-GDA-sa (**3**). Comparisons were made between GDA formed using ^16^O- and ^18^O-water. The reactions were carried out with pH 8 sodium phosphate in 1:1 MeOH-water (5 d, 30 °C). Precursor ion data are shown in Table 3. The ^13^C isotopes and K^+^ adducts were also observed. They were in accord with other findings and are not discussed. Focusing first on the ^16^O data, only a trace of unreacted GDA remained. The main reaction involved hydrolysis with small amounts of methanolysis being observed. These data are consistent with observations in the previously published paper on the structure of GDA-sa^15^ although matrix effects and the possible presence of different impurities led to minor differences between the two samples. The reaction in unlabeled water provided no evidence as to whether the hydrolysis reaction involved acyl-O or alkyl-O cleavage. Among the methanolysis products, a portion had arisen by alkyl-O cleavage, as demonstrated by the fact that carboxylic acids were the products rather than methyl esters that would have arisen by acyl-O cleavage. Proof of the presence of an alkyl-O derived carboxylic acid lay in the observation of an *m*/*z* 845.4085 ion (C_44_H_63_Na_2_O_13_^+^), which is the disodio adduct of a methanolysis-derived carboxylic acid.^20^

**Table 3:** Products of pH 8.0 Solvolysis of GDA (Na^+^ Adducts)^a^

(a) GDA Cleaved by Sodium Phosphate, pH 8.0 in 1:1 MeOH-H_2_^16^O					
observed (*m/z*)	intensity	formula	calc’d (*m/z*)	error (ppm)	notes
Unreacted GDA (1)					
791.4004	1.2e8	C_43_H_60_NaO_12_^+^	791.3977	3.48	GDA **(1**)
	Σ1.2ε8				
GDA + H_2_O					
831.3907	8.6e8	C_43_H_61_Na_2_O_13_^+^	831.3902	0.61	GDA-sa (**2**)
809.4083	2.5e9	C_43_H_62_NaO_13_^+^	809.4083	0.05	GDA-sa (**2**)
	Σ2.6e9				
GDA + MeOH					
845.4085	1.7e8	C_44_H_63_Na_2_O_13_^+^	845.4059	3.14	32-MeO-GDA-sa **7**
823.4245	7.3e8	C_44_H_64_NaO_13_^+^	823.4239	0.66	32-MeO-GDA-sa **7**
	Σ9.0e8				

(b) GDA Cleaved by Sodium Phosphate, pH 8.0 MeOH-H_2_^18^O					
Unreacted GDA (1)					
791.3995	6.2e7	C_43_H_60_NaO_12_^+^	791.3977	2.28	GDA (**1**)
	Σ6.2e7				
GDA + H_2_O					
831.3910	1.3e7	C_43_H_61_Na_2_O_13_^+^	831.3902	0.95	GDA-sa, (**2**)
809.4088	1.7e7	C_43_H_62_NaO_13_^+^	809.4083	0.66	GDA-sa, (**2**)
	Σ3.0e7				
GDA + H_2_^18^O					
835.4004	1.5e7	C_43_H_61_Na_2_O_11_^18^O_2_^+^	835.3997	0.84	GDA-sa (**2**) **+**^18^O_2_
833.3943	2.7e8	C_43_H_61_Na_2_O_12_^18^O^+^	833.3945	–0.18	GDA-sa (**2**) + ^18^O
813.4184	2.8e7	C_43_H_62_NaO_11_^18^O_2_^+^	813.4168	2.03	GDA-sa (**2**) + ^18^O_2_
811.4126	4.1e8	C_43_H_62_NaO_12_^18^O^+^	811.4125	0.11	GDA-sa (**2**) + ^18^O
	Σ7.2e8				
GDA + MeOH					
845.4069	2.4e8	C_44_H_63_Na_2_O_13_^+^	845.4059	1.23	32-MeO-GDA-sa (**7**)
825.4291	1.2e7	C_44_H_64_NaO_12_^18^O^+^	825.4282	1.14	32-MeO-GDA-sa (**7**) + ^18^O
823.4236	4.0e8	C_44_H_64_NaO_13_^+^	823.4239	–0.38	32-MeO-GDA-sa (**7**)
	Σ 6.5e8				

a See also Figure S2.

Next, opening of the macrolide ring was carried out in 1:1 H_2_^18^O/MeOH (Table 3b). Mainly, the H_2_^18^O-hydrolysis and the methanolysis products were formed but ∼10% of the unlabeled hydrolysis product was also produced. With all three products, mono- and disodio adducts were observed, providing proof that they were carboxylic acids. Unexpectedly, precursor ions were observed at *m*/*z* 813.4184 and 835.4004, reflecting seco acid into which two ^18^O atoms had been incorporated. The process by which the second ^18^O was introduced will be discussed in Section 4. For methanolysis, as in the case of the reaction carried out in unlabeled H_2_O, alkyl-O cleavage could be inferred from the presence of disodio adducts of the methanolysis products. Interestingly, the monosodio adduct of a methanolysis product was observed that also contained ^18^O (*m*/*z* 825.4291, C_44_H_64_NaO_12_^18^O^+^). This will also be discussed in Section 4. These ^18^O results provided the basis for CID studies to be undertaken so that acyl-O versus alkyl-O hydrolysis products could be determined by establishing the location of the ^18^O label.

For CID studies carried out with the FT-ICR spectrometer, data for Na^+^ adducts are presented in tables due to the dynamic range being too large to be readily viewed in spectra. On the other hand, fragment ions for NH_4_^+^ adducts are best presented as spectra rather than in tables because peaks are observed at most of the odd-numbered nominal masses over much of the spectrum. One might think these peaks to be noise but exact mass measurement reveals them to be protonated products of CID fragmentation. For cases where empirical formulas could not be assigned, they probably resulted from the presence of multiple incompletely resolved peaks of the same nominal mass. It should be noted that fragmentation of the NH_4_^+^ precursor ions yielded only NH_3_-free protonated ions. The NH_4_^+^ adducts have even-numbered nominal masses and the fragment ions were invariably odd. The FT-ICR spectrometer employed in these studies was incapable of producing useful peaks in the lower half of the spectrum.

Repetition of the published ^16^O-GDA-sa CID fragmentation study allowed comparisons to be made between them so that spurious signals could be identified.^15^ In the case of weak signals, the possibility existed that noise peaks might be present having *m*/*z* values that would have been mischaracterized as legitimate. Noise signals could though be recognized by the absence of ^13^C isotope peaks. The new fragment ion data for GDA-sa in Table 4 contained only signals that had also been observed in the first study. Eight signals were present in the original data for which empirical formulas had been assigned but structural assignments could not be proposed. Five of those signals, all weak (*m*/*z* 603.1772, 565.1022, 425.2868, 423.1356, and 413.2265), were not observed in the present study and may have been spurious in the original data set. One signal, *m*/*z* 415.1721, was present in both data sets and also in the ^18^O-labeled sample. It had been reported previously but its structure has not been assigned.^15^

**Table 4:**
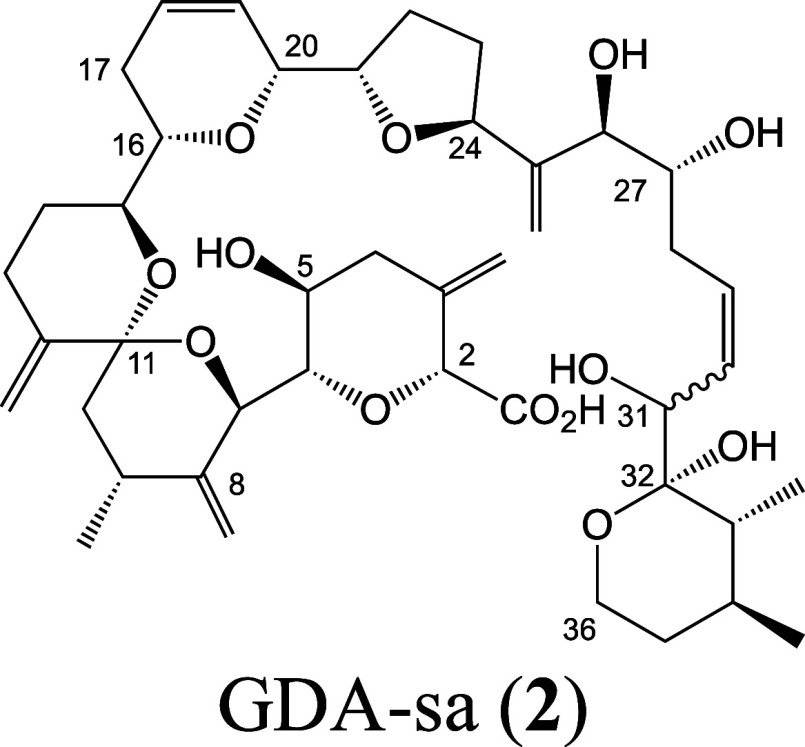
CID Fragment Ions from GDA-sa (Precursor Ion *m/z* 831, Na^+^ Adduct)^a^

observed (*m*/*z*)	intensity	formula	calculated (*m*/*z*)	error (ppm)	assignments	notes
831.3886	6.0e6	C_43_H_61_Na_2_O_13_^+^	831.3902	–0.66	C1–C36	GDA-sa (**2**) Precursor Ion (P)
813.3786	1.9e6	C_43_H_59_Na_2_O_12_^+^	813.3796	–1.28	C1–C36	P – H_2_O
791.3958	5.0e5	C_43_H_60_NaO_12_^+^	791.3977	–2.52	C1–C36	P – H_2_O - Na
773.3859	5.8e5	C_43_H_58_NaO_11_^+^	773.3871	–1.59	C1–C36	P – 2H_2_O
765.4172	1.8e6	C_42_H_62_NaO_11_^+^	765.4184	–1.61	C2–C36	**5**, P – CO_2_, Grob–Wharton
747.4065	2.0e6	C_42_H_60_NaO_10_^+^	747.4079	–1.83	C2–C36	P – CO_2_ - H_2_O, Grob–Wharton
729.3961	1.2e6	C_42_H_58_NaO_9_^+^	729.3973	–1.65	C2–C36	P – CO_2_ – 2H_2_O, Grob–Wharton
565.2763	1.8e6	C_31_H_42_NaO_8_^+^	565.2772	–1.57	C2–C27	P – CO_2_, Grob–Wharton
537.2814	6.0e5	C_30_H_42_NaO_7_^+^	537.2823	–1.63	C2–C26	P – CO_2_, Grob–Wharton
495.2345	1.2e6	C_27_H_36_NaO_7_^+^	495.2353	–1.66	C5–C27	Grob–Wharton
467.2397	7.2e5	C_26_H_36_NaO_6_^+^	467.2404	–1.52	C5–C26	P – CO_2_, Grob–Wharton
431.2398	1.8e7	C_23_H_36_NaO_6_^+^	431.2404	–1.41	C17–C36	RDA Tail
429.2241	1.6e6	C_23_H_34_NaO_6_^+^	429.2248	–1.54	C17–C36	RDA Tail
423.1384	4.2e7	C_20_H_25_Na_2_O_7_^+^	423.1390	–1.46	C1–C16	RDA Head
415.1721	3.8e6	C_21_H_28_NaO_7_^+^	415.1727	–1.50	C1–C16, Me ester	RDA Head
413.2292	3.5e7	C_23_H_34_NaO_5_^+^	413.2298	–1.56	C17–C36	RDA Tail
401.1565	4.3e6	C_20_H_26_NaO_7_^+^	401.1571	–1.43	C1–C16	RDA Head
395.2187	1.6e7	C_23_H_32_NaO_4_^+^	395.2193	–1.47	C17–C36	RDA Tail
385.1980	3.3e6	C_21_H_30_NaO_5_^+^	385.1985	–1.41	unassigned	
377.2082	2.2e6	C_23_H_30_NaO_3_^+^	377.2087	–1.37	C17–C36	RDA Tail
367.1875	1.8e6	C_21_H_28_NaO_4_^+^	367.1880	–1.31	unassigned	
357.1667	1.9e7	C_19_H_26_NaO_5_^+^	357.1672	–1.52	C2–C16	RDA Head
351.1174	1.2e6	C_17_H_21_Na_2_O_5_^+^	351.1179	–1.39	C1–C13	
349.1769	7.7e5	C_21_H_26_NaO_3_^+^	349.1774	–1.48	unassigned	
287.1250	3.6e6	C_15_H_20_NaO_4_^+^	287.1254	–1.32	C5–C16	RDA Head
255.0601	6.4e5	C_11_H_13_Na_2_O_4_^+^	255.0604	–1.07	unassigned	
233.1145	1.0e6	C_12_H_18_NaO_3_^+^	233.1148	–1.35	C27–C36	Grob–Wharton
231.0989	1.5e6	C_12_H_16_NaO_3_^+^	231.0992	–1.15	C17–C27	Grob–Wharton

a XGDA-sa formed by sodium phosphate, pH 8.0 in 1:1 MeOH-H_2_O. See also Figure S3.

The ^18^O CID data in Table 5 in conjunction with the ^16^O data in Table 4 became a powerful tool for making assignments and consequently facilitated elucidation of mechanistic details of the hydrolysis reaction. Alkyl-O cleavage was mainly observed, giving an *m*/*z* 423 head fragment which lacked ^18^O. A small amount of acyl-O cleavage occurred, yielding the ^18^O-labeled *m*/*z* 425 head fragment. With alkyl-O cleavage, the tail fragment would contain the ^18^O label. Head and tail regions of GDA are defined on the basis of the biosynthesis.^21^ C1–C16 and appendages are defined as the “head” and C17–C36 and appendages are defined as the “tail”. They are abbreviated as “H” and “T”.

**Table 5:** CID Fragment Ions from ^18^O-GDA-sa (Precursor Ion: *m/z* 833, Na^+^ Adduct)^a^

observed, *m*/*z*	intensity	formula	calculated, *m*/*z*	error (ppm)	assignment	notes
833.3927	9.1e5	C_43_H_61_Na_2_O_12_^18^O^+^	833.3942	–2.10	C1–C36	Precursor Ion, GDA-sa **2** with ^18^O
566–832	Not obs					
565.2761	6.5e5	C_31_H_42_NaO_8_^+^	565.2772	–1.93	C2–C27	Grob–Wharton - CO_2_
435.2482	1.6e6	C_23_H_36_NaO_4_^18^O_2_^+^	435.2489	–1.61	C17–C36	RDA Tail + ^18^O_2_
433.2440	3.9e6	C_23_H_36_NaO_5_^18^O^+^	433.2447	–1.51	C17–C36	RDA Tail + ^18^O
433.2326	2.7e6	C_23_H_34_NaO_4_^18^O_2_^+^	433.2332	–1.50	C17–C36	RDA Tail + ^18^O_2_
425.1427	6.8e5	C_20_H_25_Na_2_O_6_^18^O^+^	425.1433	–1.32	C1–C16	RDA Head C1-^18^O
423.1384	1.3e7	C_20_H_25_Na_2_O_7_^+^	423.1390	–1.46	C1–C16	RDA Head
415.2335	2.1e6	C_23_H_34_NaO_4_^18^O^+^	415.2341	–1.42	C17–C36	RDA Tail, C32-^18^O
415.1721	2.3e6	C_21_H_28_NaO_7_^+^	415.1727	–1.50	C1–C16, Me ester	RDA Head
413.2292	1.1e7	C_23_H_34_NaO_5_^+^	413.2298	–1.56	C17–C36	RDA Tail
401.1565	1.8e6	C_20_H_26_NaO_7_^+^	401.1571	–1.43	C1–C16	RDA Head
395.2187	7.1e6	C_23_H_32_NaO_4_^+^	395.2193	–1.47	C17–C36	RDA Tail
385.1980	1.6e6	C_21_H_30_NaO_5_^+^	385.1985	–1.41	unassigned	
377.2081	1.4e6	C_23_H_30_NaO_3_^+^	377.2087	–1.50	C17–C36	RDA Tail
367.1875	8.6e5	C_21_H_28_NaO_4_^+^	367.1880	–1.31	unassigned	
357.1667	8.7e6	C_19_H_26_NaO_5_^+^	357.1672	–1.52	C2–C16	RDA Head
287.1250	1.6e6	C_15_H_20_NaO_4_^+^	287.1254	–1.32	C5–C16	RDA Head
233.1145	6.1e5	C_12_H_18_NaO_3_^+^	233.1148	–1.35	C27–C36	Grob–Wharton
231.0988	5.2e5	C_12_H_16_NaO_3_^+^	231.0992	–1.58	C17–C27	**11**

a GDA-sa formed by sodium phosphate, pH 8.0 in 1:1 MeOH-H_2_^18^O. See also Figure S4.

**Figure 2:**
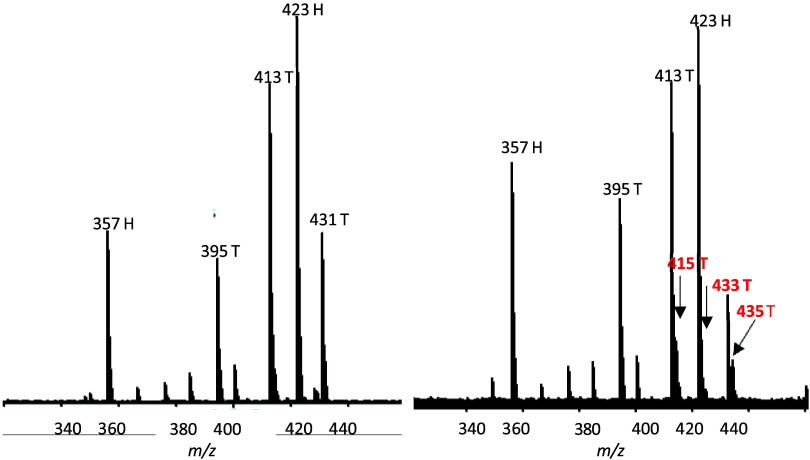
CID fragmentation spectra of ^18^O-labeled and unlabeled GDA-sa on (right and left, respectively). Head and tail assignments are indicated by “H” and “T”. Signals bearing ^18^O labels are indicated in red. Exact masses and other data acquired with the FT-ICR spectrometer are listed in Tables 4 and 5.

The data contained in Tables 4 and 5 can be comprehended more readily by side-by-side visual comparison of the *m*/*z* 340–440 region of the labeled and unlabeled spectra. The ^18^O and ^16^O spectra are on the right and left, respectively, in Figure 2. The ^16^O sample was more concentrated than the ^18^O, producing a higher signal-to-noise. Head fragment ions lacking incorporation of ^18^O label were observed (*m*/*z* 423 and 357) in both the ^16^O and ^18^O spectra. Tail fragment ions in the ^16^O spectrum were observed at *m*/*z* 431, 413, and 395. Tail fragment ions bearing ^18^O labels were observed at *m*/*z* 415 and 433 and are indicated in red. They were not fully resolved on the UPLC-MS/MS instrument from the more intense unlabeled signals at *m*/*z* 413, 423, and 431. Despite this deficiency, they gave satisfactory exact mass values on the FT-ICR spectrometer. Most of the isotopic label had been carried along during loss of H_2_O molecules to form these ions. The *m*/*z* 395 ion showed no indication of having a comparable isotopic signal at *m*/*z* 397. Isotopic label, if any were still present, was below the level of detection.

**Figure 3:**
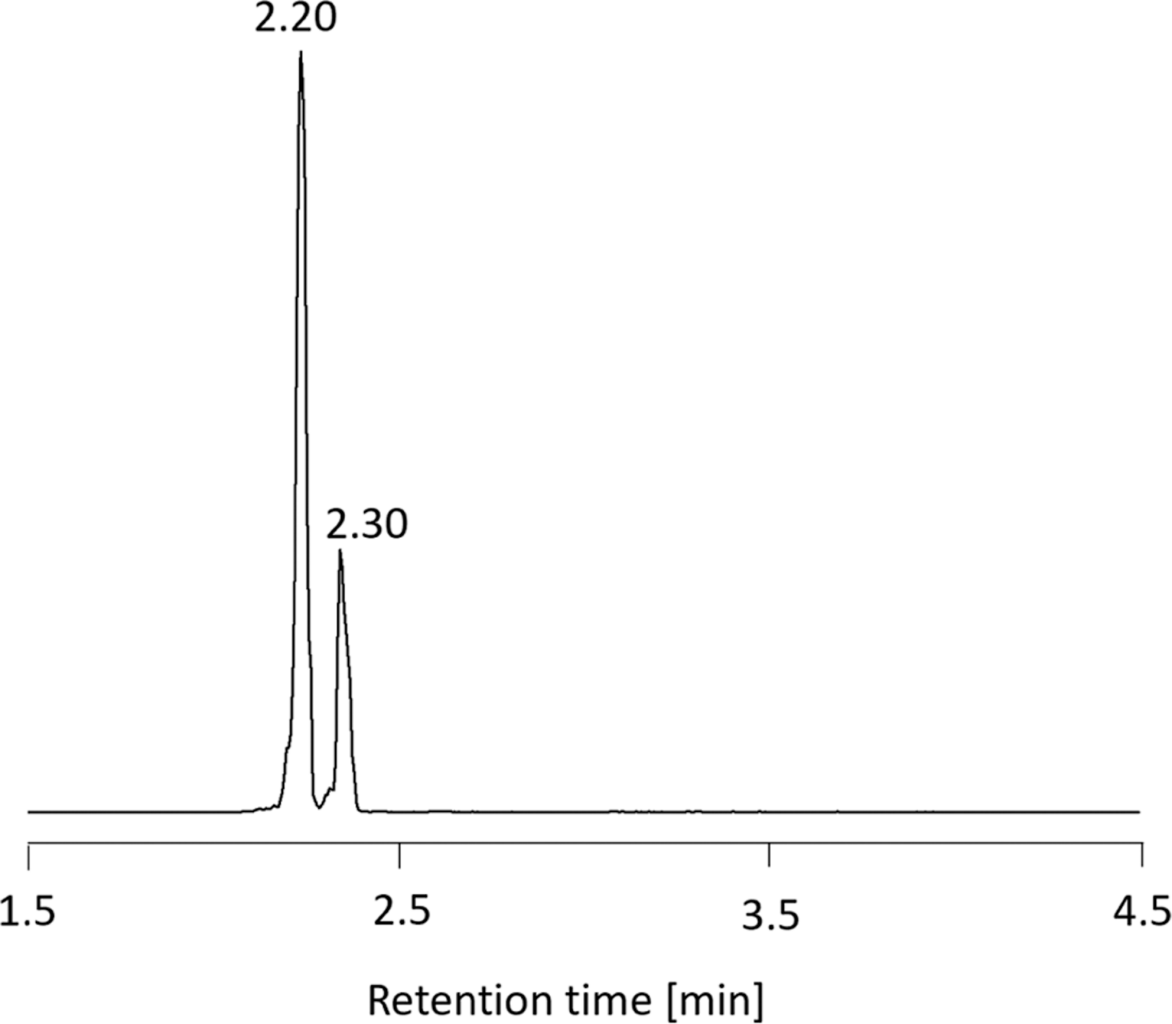
Chromatogram of ^18^O-GDA-sa (SRM, sum of Na^+^ and NH_4_^+^ adducts).

**Figure 4:**
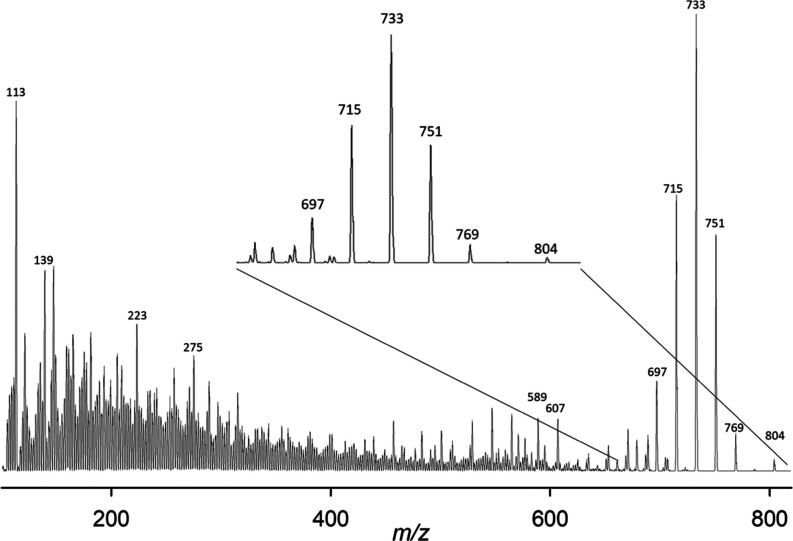
CID mass spectrum of NH_4_^+^ adducts of the 2.20 min peak in the SRM chromatogram of ^16^O-GDA-sa. Note inset *x*-axis expansion of upper mass region.

**Figure 5:**
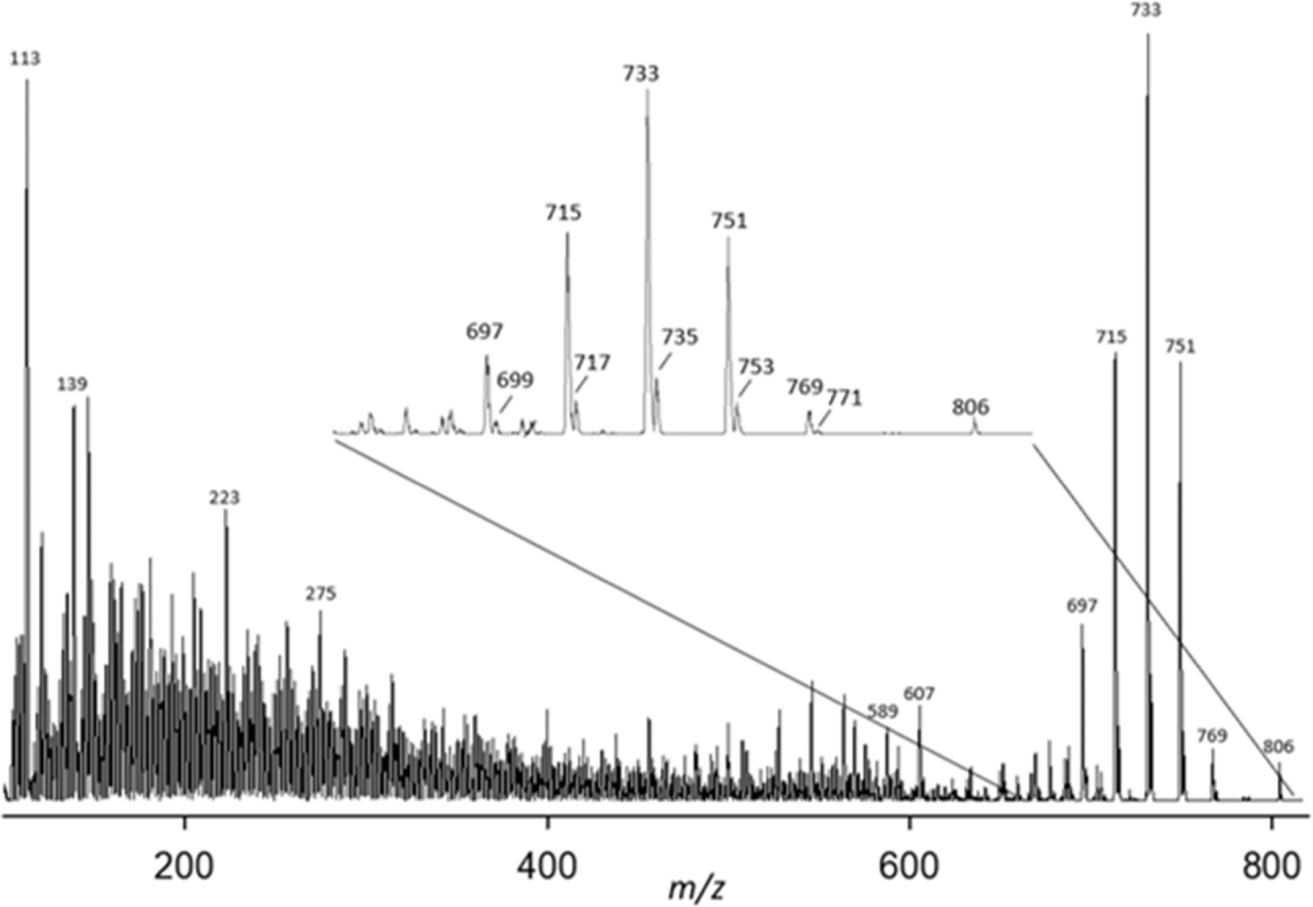
CID mass spectrum of NH_4_^+^ adducts of the 2.20 min peak in the SRM chromatogram of ^18^O-GDA-sa. Note inset expansion of upper mass region.

**Figure 6:**
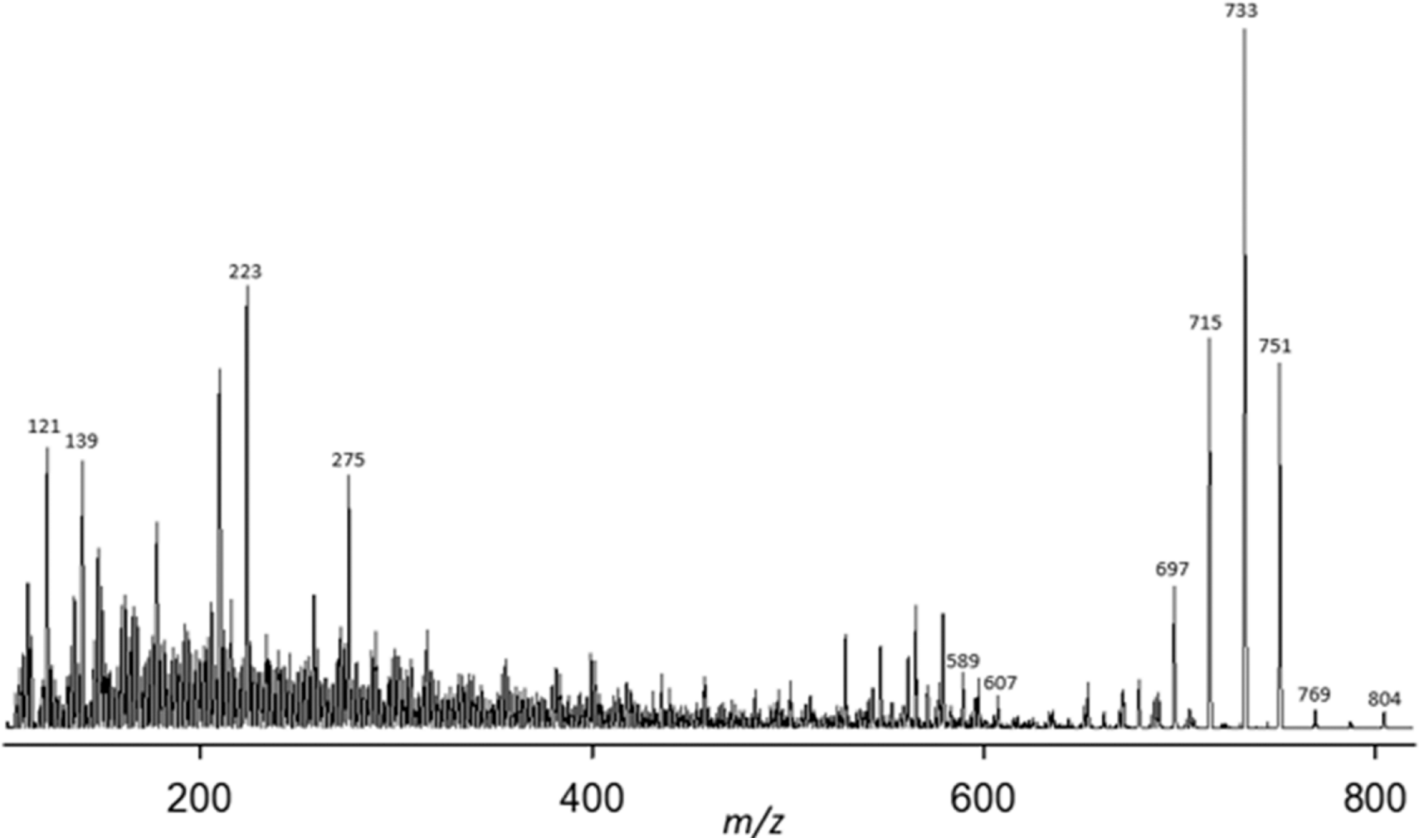
CID mass spectrum of the 2.30 min peak in the SRM chromatogram of ^16^O-GDA-sa.

CID fragmentation of the ^18^O seco acids was also studied by UPLC-MS/MS (Figures 3–6). While the instrument lacked the high-resolution benefit of the FT-ICR spectrometer for establishing empirical formulas by exact mass measurement, it offered the additional dimension of liquid chromatography to the analysis. The C18 reversed-phase column divided the Na^+^ salts of the ^18^O-labeled seco acids into 2–3 peaks, a large one eluting at 3.17 min followed by one of intermediate size at 3.37 min and in some cases a small one at 3.46 min. The previously published chromatogram of the ^16^O seco acid was similar but comprised only two peaks.^15^ Fragmentation data for the first two peaks for the ^16^O and ^18^O isotopic samples are provided in Table 6. The S/N values of fragment ions in the small third peak were too low to be of value and are not reported. Some of the previously reported ^16^O data has now been reinterpreted, aided by the ^18^O data. In particular, an assignment problem existed with the *m*/*z* 231.0988 ion (C_12_H_16_NaO_3_^+^) which was observed in both the FT-ICR and triple quadrupole spectra. Although previously assigned as being C2–C10,^15^ it is more likely to be C27–C36, formed without inclusion of ^18^O.

**Figure 7:**
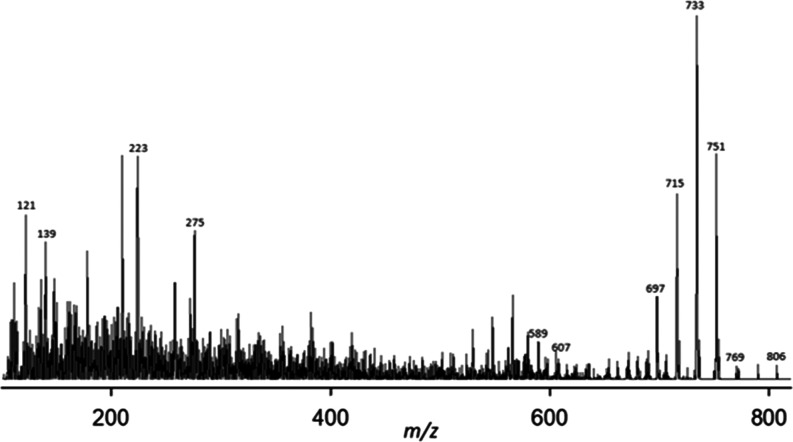
CID mass spectrum of the 2.30 min peak in the SRM chromatogram of ^18^O-GDA-sa.

**Table 6:** CID Spectra of ^16^O- and ^18^O-GDA-sa (Na^+^ Adducts)^a^

	*m/z* 809, ^16^O		*m/z* 811, ^18^O				
	3.17 min	3.37 min	3.17 min	3.37 min			
precursor *m/z*	intensity	intensity	intensity	intensity	formula	assignment	notes
811	ND	ND	1.1e6	1.3e6	C_43_H_62_NaO_12_^18^O^+^	C1–C36	^18^O Precursor (P’)
809	5.2e6	3.3e6	ND	ND	C_43_H_62_NaO_13_^+^	C1–C36	^16^O Precursor (P)
793	ND	ND	3.5e5	8.5e4	C_43_H_60_NaO_11_^18^O^+^	C1–C36	P’ – H_2_O
791	1.9e6	6.1e5	3.2e5	1.8e5	C_43_H_60_NaO_12_^+^	C1–C36	P – H_2_O
773	1.1e6	ND	ND	ND	C_43_H_58_NaO_11_^+^	C1–C36	P – 2H_2_O
767	ND	ND	4.9e6	8.8e5	C_42_H_62_NaO_10_^18^O^+^	C2–C36	P’ – CO_2_
765	1.5e7	7.7e6	3.5e4	ND	C_42_H_62_NaO_11_^+^	C2–C36	P – CO_2_
749	ND	ND	7.7e5	ND	C_42_H_60_NaO_9_^18^O^+^	C2–C36	P’ – CO_2_ – H_2_O
747	3.3e6	1.0e6	5.1e5	3.2e5	C_42_H_60_NaO_10_^+^	C2–C36	P – CO_2_ – H_2_O
731	ND	ND	1.4e5	ND	C_42_H_58_NaO_8_^18^O^+^	C2–C36	P’ – CO_2_ – 2H_2_O
729	1.5e6	ND	3.8e5	ND	C_42_H_58_NaO_9_^+^	C2–C36	P – CO_2_ – 2H_2_O
697	ND	ND	3.7e5	2.9e5	C_38_H_56_NaO_9_^18^O^+^	C5–C36	RDA
695	1.3e6	6.2e5	ND	ND	C_38_H_56_NaO_10_^+^	C5–C36	RDA
609	5.7e5	ND	1.8e5	ND	C_32_H_42_NaO_10_^+^	C1–C27	
565	1.7e6	ND	5.3e5	ND	C_31_H_42_NaO_8_^+^	C2–C27	
537	5.3e5	ND	1.7e5	3.5e5	C_30_H_42_NaO_7_^+^	C2–C26	Grob–Wharton
433	ND	ND	3.5e6	2.0e6	C_23_H_36_NaO_5_^18^O^+^	C17–C36	RDA Tail
431	1.2e7	5.2e6	5.3e5	1.6e5	C_23_H_36_NaO_6_^+^	C17–C36	RDA Tail
415	ND	ND	5.3e5	1.2e5	C_23_H_34_NaO_4_^18^O^+^	C17–C36	RDA Tail
413	3.9e6	9.5e5	8.1e5	4.0e5	C_23_H_34_NaO_5_^+^	C17–C36	
401	2.7e6	1.5e6	9.0e5	6.8e5	C_20_H_26_NaO_7_^+^	C1–C16	RDA Head
395	1.5e6	ND	3.6e5	ND	C_23_H_32_NaO_4_^+^	C17–C36	RDA Tail
357	3.4e6	1.8e6	7.9e5	9.9e5	C_19_H_26_NaO_5_^+^	C2–C16	RDA Head
287	5.5e5	3.3e5	2.6e5	ND	C_15_H_20_NaO_4_^+^	C5–C16	
251	5.1e5	ND	ND	ND	C_12_H_20_NaO_4_^+^	C27–C36	
231	8.2e5	ND	4.4e5	ND	C_12_H_16_NaO_3_^+^	C17–C27	

a Empirical formulas and carbon assignments inferred from the FT-ICR data in Tables 4 and 5. ND = Not Detected.

It should be noted that the UPLC-MS/MS CID data in Table 6 were acquired with monosodio adducts whereas the high-resolution FT-ICR CID data presented in Tables 4 and 5 had been collected for the disodio species. The two types of adducts complement one another. The disodio adducts provide unambiguous assignments for fragment ions containing carboxyl head groups. Only three fragment ions containing ^18^O were observed in the FT-ICR CID spectrum of the disodio adduct whereas in the triple quadrupole spectrum of the monosodio adducts of ^18^O-GDA-sa seven fragment ions could be assigned as containing ^18^O. As discussed further below, the isotopic data are consistent with all ^18^O-containing fragment ions being derived from the tail region of GDA-sa.

The Na^+^ and NH_4_^+^ adducts of the ^16^O and ^18^O samples of GDA-sa were examined using UPLC-MS/MS. The ^16^O SRM chromatogram had been recently reported.^15^ The ^18^O chromatogram was now obtained similarly (Figure 3), showing a large peak at 2.20 min and a small one at 2.30 min. The CID spectra of the NH_4_^+^ adducts of the ^16^O and ^18^O samples (Figures 4–7) were marred by nonspecific peaks at each of the odd-numbered masses which were particularly intense in the lower mass region. A few of the most intense low-mass peaks rose out of this background noise sufficiently to have diagnostic value.

### 3.2 Ring-Opening of GDA with Anhydrous Methanolic Na_2_CO_3_ and NH_3_

Cleavage products formed by treatment of GDA with anhydrous methanolic Na_2_CO_3_ were analyzed using the FT-ICR spectrometer. Data for mono- and disodio adducts of GDA-sa (and small amounts of iso-GDA-sa) formed by hydrolysis (C_43_H_60_NaO_12_^+^) and methanolysis (C_44_H_64_NaO_13_^+^) are presented in Table 7a. The mono- and disodio adducts of a carboxylic acid having the same empirical formula (C_43_H_60_O_12_) as GDA are shown. Assignment of the structure of this carboxylic acid as goniodomic acid (**4**) is made in Section 4. The most intense peaks (*m*/*z* 809.4083 and 831.3902) were for the mono- and disodio adducts of the seco acid(s) formed by hydrolysis, *in spite of the cleavage reaction having been carried out under anhydrous conditions*. The mono- and disodio adducts of the methanolysis product were observed but at peak intensities much lower than those of the GDA-sa. The disodio ions are indicative of carboxylic acids^20^ which would have arisen by alkyl-O cleavage of the ester linkage.

**Table 7:** Seco Acids Formed from GDA under Anhydrous Conditions^a^

(a) Methanolic Na_2_CO_3_					
observed (*m/z*)	intensity	formula	calculated (*m/z*)	error (ppm)	assignment
813.3799	2.6e6	C_43_H_59_Na_2_O_12_^+^	813.3796	0.30	Goniodomic Acid (**4**)
791.3976	1.9e6	C_43_H_60_NaO_12_^+^	791.3977	–0.11	Goniodomic Acid (**4**)
	Σ4.5e6				
831.3902	1.2e7	C_43_H_61_Na_2_O_13_^+^	831.3902	0.02	GDA-sa (**2**)
809.4083	1.4e7	C_43_H_62_NaO_13_^+^	809.4083	0.08	GDA-sa (**2**)
	Σ2.6e7				
845.4057	1.6e6	C_44_H_63_Na_2_O_13_^+^	845.4059	–0.13	32-MeO-GDA-sa (**7**)
823.4240	1.6e6	C_44_H_64_NaO_13_^+^	823.4239	0.13	32-MeO-GDA-sa (**7**)
	Σ3.2e6				

(b) Methanolic NH_3_. Polar Fraction					
observed (*m/z*)	intensity	formula	calculated (*m/z*)	error (ppm)	assignment
813.3809	1.1e6	C_43_H_59_Na_2_O_12_^+^	813.3796	1.56	Goniodomic Acid (**4**)
791.3990	5.1e6	C_43_H_60_NaO_12_^+^	791.3977	1.64	Goniodomic Acid (**4**)
	Σ6.2e6				
831.3908	6.4e6	C_43_H_61_Na_2_O_13_^+^	831.3902	0.77	GDA-sa (**2**)
809.4088	8.9e6	C_43_H_62_NaO_13_^+^	809.4083	0.64	GDA-sa (**2**)
	Σ1.5e7				
845.4071	4.1e7	C_44_H_63_Na_2_O_13_^+^	845.4059	–1.38	32-MeO-GDA-sa (**7**)
823.4244	4.6e7	C_44_H_64_NaO_13_^+^	823.4239	0.59	32-MeO-GDA-sa (**7**)
	Σ9.7e7				

(c) Methanolic NH_3_. Nonpolar Fraction					
observed (*m/z*)	intensity	formula	calculated (*m/z*)	error (ppm)	assignment
823.4236	1.0e8	C_44_H_64_NaO_13_^+^	823.4239	–0.38	Me ester of **2**
791.3983	3.1e6	C_43_H_60_NaO_12_^+^	791.3977	0.74	GDA (**1**)
	Σ1.0e8				

a See also Figure S5.

Products resulting from treatment of GDA with anhydrous, methanolic NH_3_ followed by preparative HPLC (C18, ACN-H_2_O gradient) were also examined. Mono- and disodio adducts of goniodomic acid **4**, GDA-sa (**2**) and 32-MeO-GDA-sa (**7**) were observed in the polar fraction (Table 7b). **7** was the major component of the mixture. The presence of GDA-sa is ascribed to partial hydrolysis of the oxirane ring during HPLC purification. The nonpolar fraction contained methylated GDA-sa (*m*/*z* 823.4236, C_44_H_64_NaO_13_^+^) plus ∼3% of unreacted GDA. The CID spectrum of the *m*/*z* 823 ion (Table 8) contained an intense *m*/*z* 415.1727 fragment ion by which the *m*/*z* 415.1727 could be assigned as that of the Me ester of the C1–C16 head fragment. Unmethylated tail fragments were observed at *m*/*z* 431.2405, 413.2299, and 395.2193. Sodiated ions arose from adventitious Na^+^. Nitrogen-containing fragment ions were not detected.

**Table 8:** CID Fragment Ions from the Methyl Ester of Seco Acid **2** (*m/z* 823) Formed by Reaction of GDA with Anhydrous, Methanolic NH_3_^a^

observed (*m/z*)	intensity	formula	calculated (*m/z*)	error (ppm)	assignment
823.4236	4.5e6	C_44_H_64_NaO_13_^+^	823.4239	–0.09	precursor ion, Me ester, mainly of **2**
805.4135	2.0e6	C_44_H_62_NaO_12_^+^	805.4133	0.19	Me ester – H_2_O
787.4027	4.2e5	C_44_H_60_NaO_11_^+^	787.4028	–0.11	Me ester – 2H_2_O
623.2826	4.9e5	C_33_H_44_NaO_10_^+^	623.2827	–0.11	C1–C27, RDA Head
431.2405	3.3e6	C_23_H_36_NaO_6_^+^	431.2404	0.21	C17–C36, RDA Tail
415.1727	1.1e8	C_21_H_28_NaO_7_^+^	415.1727	–0.06	C1–C16, Me Ester
413.2299	3.8e6	C_23_H_34_NaO_5_^+^	413.2298	0.13	C17–C36, Tail – H_2_O
395.2193	2.3e6	C_23_H_32_NaO_4_^+^	395.2193	0.05	C17–C36, Tail −2H_2_O
359.1466	7.5e5	C_18_H_24_NaO_6_^+^	359.1465	0.25	unassigned
343.1516	3.5e6	C_18_H_24_NaO_5_^+^	343.1516	0.02	unassigned

a See also Figure S6.

## 4 Discussion

### 4.1 Alkyl-O versus Acyl-O Cleavage of Macrolide Ring

**Scheme 2:**
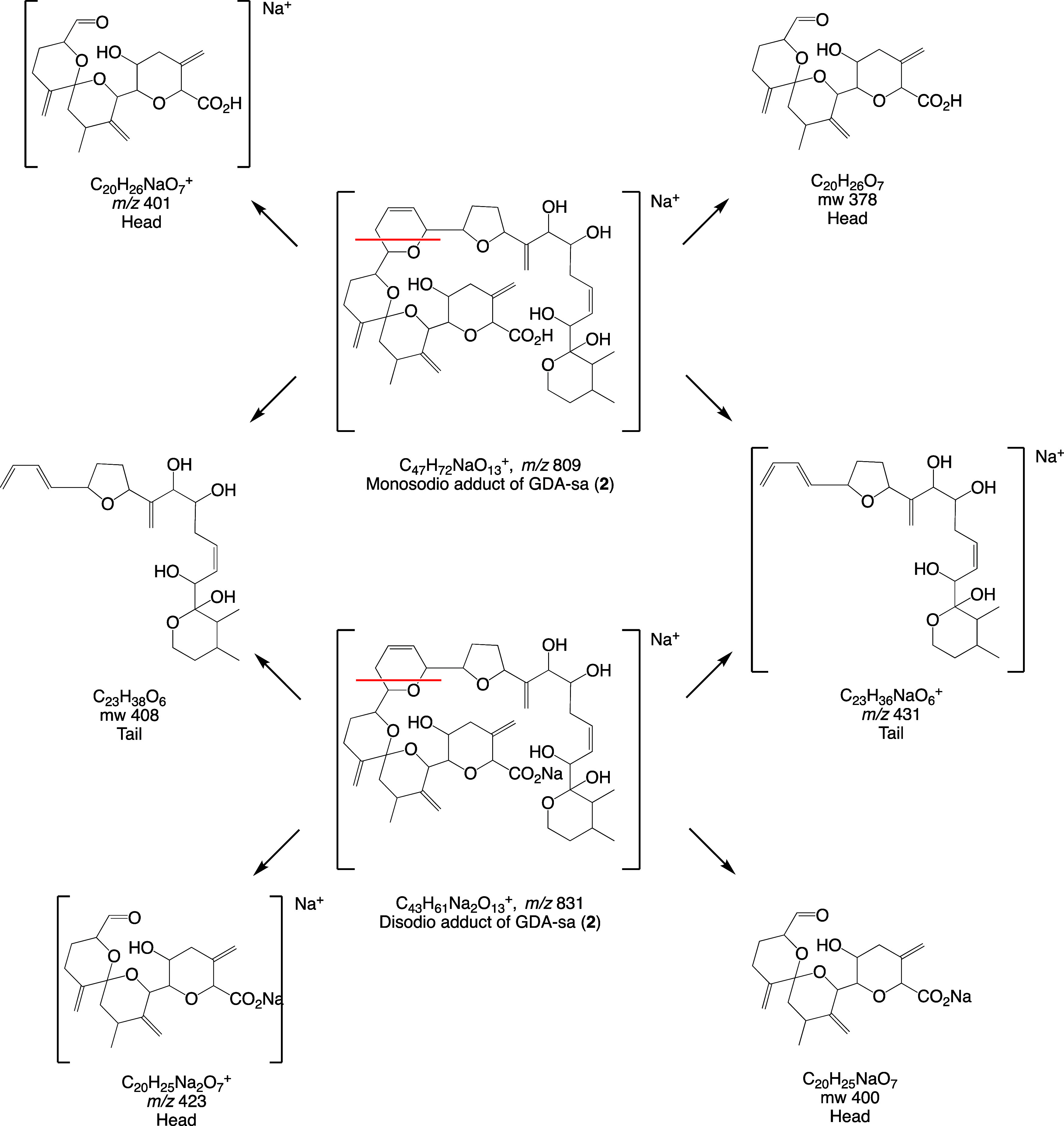
RDA Fragmentation of the Mono- and Disodio Adducts of GDA-sa, Upper and Lower Sectors of the Scheme, Respectively Red lines indicate RDA cleavage site.

The studies of ^18^O incorporation show that cleavage of the macrolide ring occurs mainly by alkyl-O fragmentation of the ester linkage. The site of ^18^O-incorporation was established by mass spectrometry, relying in particular on retro-Diels–Alder (RDA) fragmentation. RDA methodology has been employed extensively for structural analysis of natural products.^22−26^ RDA fragments are observed when compounds contain cyclohexene and oxene rings. High resolution CID spectra of GDA-sa were acquired on the *m*/*z* 831.3886 ion (disodio adduct; C_43_H_61_Na_2_O_13_^+^). The RDA process yielded head and tail ions observed at *m*/*z* 423.1384 (C_20_H_25_Na_2_O_7_^+^) and 431.2398 (C_23_H_36_NaO_6_^+^), respectively (Table 4 and Scheme 2). The fragment ions reflect the occurrence of tandem fragmentation processes with one having the positive charge placed on the head fragment and the other having the positive charge placed on the tail. These fragment ions are consistent with the previously assigned structure of GDA-sa.^15^

When conversion of GDA to seco acids was carried out in ^18^O-water, disodio ion spectra (FT-ICR) showed that the ^18^O label had been incorporated into the seco acids (*m*/*z* 811.4126, C_43_H_62_NaO_12_^18^O^+^ and *m*/*z* 833.3943, C_43_H_61_Na_2_O_12_^18^O^+^, Table 3b). CID spectra acquired on the *m*/*z* 833.3943 ion (disodio adduct) showed that the *m*/*z* 431.2392 C17–C36 tail fragment ion from GDA-sa was labeled with ^18^O, raising the *m*/*z* to 433.2440 (Table 5). Three sequential dehydration steps occurred with *m*/*z* 433.2440. The first showed 84% loss of ^18^O label, giving a 6:1 mixture of *m*/*z* 413.2292 (C_23_H_34_NaO_5_^+^) and 415.2335 (C_23_H_34_NaO_4_^18^O^+^). The remaining ^18^O label was lost in the second dehydration step. No labeling was seen in the head fragment. Fragmentation studies with the monosodio adduct (UPLC-MS/MS) gave confirmatory results (Table 6). NH_4_^+^ adducts of GDA-sa were also examined but showed no evidence of RDA fragmentation. The qualitative results are of significance but quantitative interpretations of the ^18^O data must be made with caution because substantial amounts of ^18^O label may have been lost by exchange during HPLC-UV and UPLC-MS analyses.

**Scheme 3:**
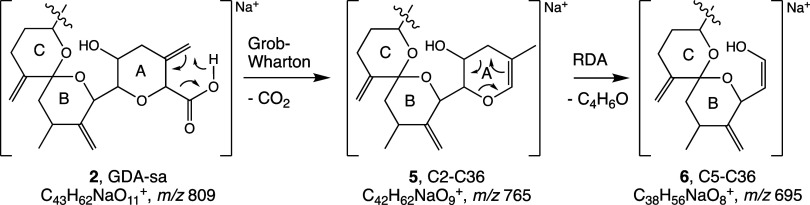
Grob–Wharton Decarboxylation of Sodio Adduct of GDA-sa (**2**) Yields Oxene **5** RDA conversion of **5** to **6** only observed with the UPLC-MS/MS.

Decarboxylation of ^18^O-labeled seco acids is another approach for distinguishing alkyl-O from acyl-O cleavage of macrolide rings since acyl-O cleavage will place the ^18^O label in the departing CO_2_ molecule. The approach is limited to situations where seco acids readily undergo decarboxylation. This condition is met by seco acids **2** and **3** because they have a β,γ-double bond that will cause decarboxylation to occur by Grob–Wharton pericyclic fragmentation as shown in Scheme 3.^26,27^ Decarboxylation of the seco acids was observed for both chromatographic peaks (Scheme 3) and both the mono- and disodio adducts of seco acids **2** and **3**. In all cases, monosodio adduct **5** (C_42_H_62_NaO_11_^+^, *m*/*z* 765.4172) was formed (Table 4). With UPLC-MS/MS (Table 6), the oxene ring of **5** (*m*/*z* 765) underwent RDA fragmentation to yield the C5–C36 fragment ion **6** (*m*/*z* 695). Loss of H_2_O, presumably from C5, was a competing reaction. With the FT-ICR spectrometer, only loss of H_2_O was observed. The basis for this difference is not known.

When conversion of GDA to seco acids was carried out in ^18^O-water, the CID spectra of the disodio adducts (FT-ICR) showed that one ^18^O atom had been incorporated into the seco acids (*m*/*z* 811.4126, C_43_H_62_NaO_12_^18^O^+^, and *m*/*z* 833.3934, C_43_H_61_NaO_12_^18^O^+^, Table 3b). The CID spectrum of the *m*/*z* 833.3943 ion showed the RDA tail fragment to be labeled, i.e., the *m*/*z* had been raised from *m*/*z* 431.2404 to 433.2440 while the RDA head fragment remained unchanged. With UPLC-MS/MS, CID decarboxylation of the monosodio adduct of labeled GDA-sa showed retention of ^18^O in the product ion (*m*/*z* 767, C_42_H_62_NaO_10_^18^O^+^, Table 6), indicating that the carboxyl group had not borne the ^18^O label and established that ring-opening of GDA had resulted from alkyl-O cleavage. Losses of 40 and 73% of the ^18^O were observed in two subsequent dehydration steps. This experiment had been carried out initially with the 3.17 min chromatographic peak but similar results were obtained with the 3.37 min peak. At this point, alkyl-O cleavage had been unambiguously established. Decarboxylation was not observed with the NH_4_^+^ adduct of GDA-sa (Figures 4–7).

An ^18^O atom is incorporated into the carboxyl group during acyl-O cleavage of esters, whereas the ^18^O is inserted into the alkyl group during alkyl-O cleavage. It should be pointed out that, once formed, the carboxyl groups of GDA-sa and iso-GDA-sa are not subject to further isotopic exchange under the alkaline conditions employed in the present studies. This can be a point of confusion because exchange will occur in the carboxyl group under acidic conditions.^28,29^

### 4.2 Formation of the Goniodomic Acid during Opening of the Macrolide Ring

**Scheme 4:**
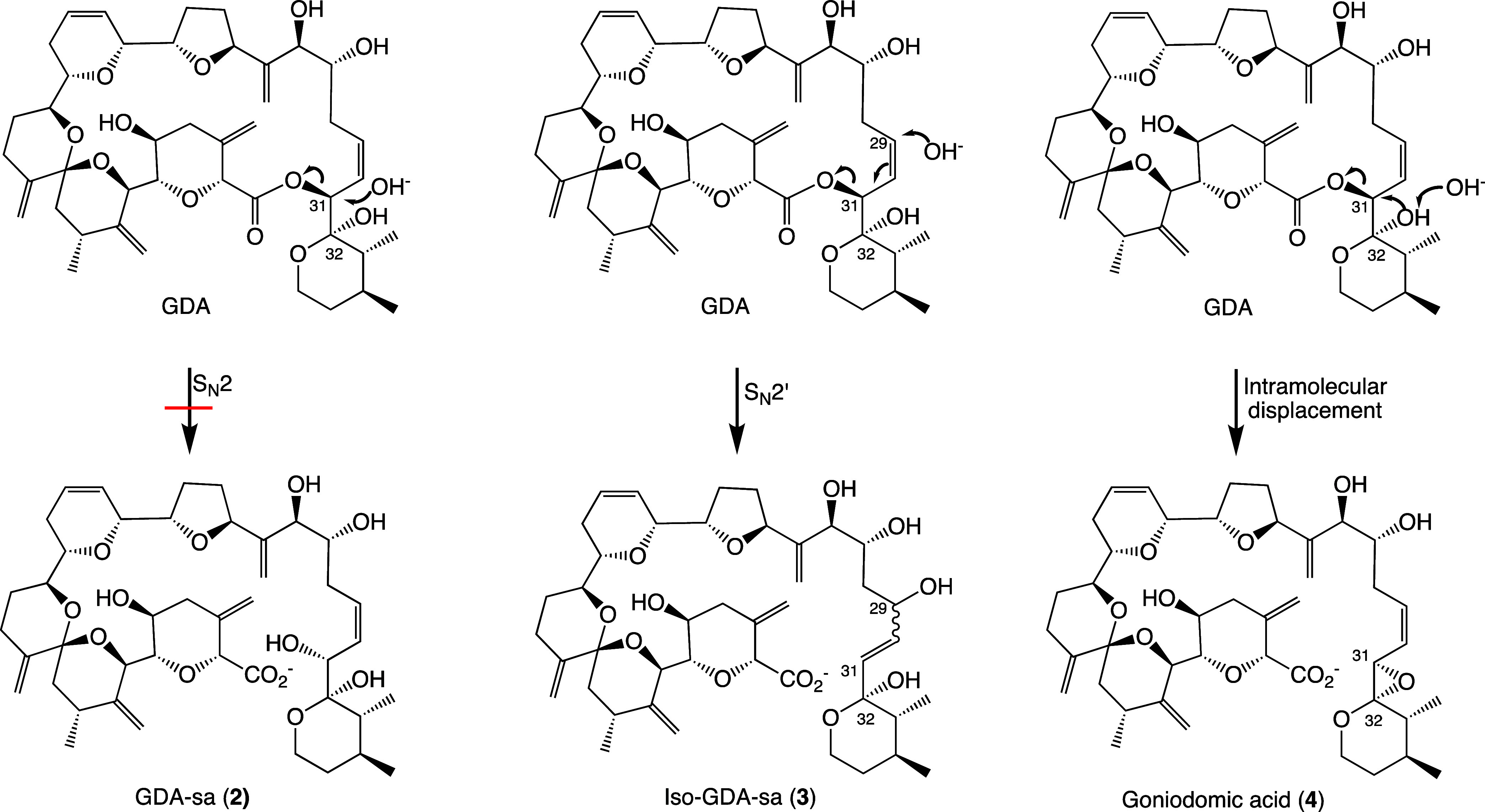
Pathways for Alkyl-O Ring-Opening of the Macrolide Ring of GDA Direct attack at C31 (S_N_2), allylic attack at C29 (S_N_2′), and intramolecular attack of the C32–O^–^ on C31.

Alkyl-O cleavage of the macrocyclic ring might occur by three routes: (1) S_N_2 displacement at C31, (2) allylic attack at C29 and (3) intramolecular displacement by the C32 hydroxy group (Scheme 4). The third pathway creates an oxirane ring. Direct S_N_2 displacement at C31 is unlikely due to steric constraints. In our recent paper,^15^ allylic attack was proposed to be the primary cleavage process with the potential involvement of an oxirane intermediate being a secondary pathway. At that time, experimental evidence had not come forth to support involvement of an oxirane intermediate. We speculated that the large chromatographic peak contained iso-GDA-sa (**3**) and the smaller peak GDA-sa (**2**). Predicted polarities of **2** and **3** had suggested that **3** would elute faster than **2** from reversed-phase HPLC columns. That prediction overlooked the potential hydrogen bond between 27-OH and 29-OH of **3** which would increase its lipophilicity such that these assignments should be reversed. In support of reassignment, the kinetics of the two pathways should favor formation of **2**.

The DFT calculations by Hess and Smentek described in the Introduction indicated that formation of the oxirane ring would be facilitated by the antiperiplanar orientation of the hydroxy groups on C31 and C32.^17^ The preferred axial orientation of the C32 hydroxy group provides a clear pathway for backside displacement of the ester oxy atom on C31. The conformational requirements for allylic attack are more demanding due to the steric constraints of the allylic moiety resulting from the *Z* configuration of the C29–C30 double-bond and those of the macrolide ring itself.^30^ This assertion is supported by data on S_N_2 and S_N_2′ nucleophilic attack on butadiene epoxide presented in Section 4.6.

Our initial studies had provided no evidence for the formation of oxirane species but new experimental results give strong support for the Hess–Smentek proposal^17^ with intramolecular displacement being the dominant pathway (Schemes 1 and 4). The resulting oxirane product **4**, herein named goniodomic acid, is a transient species. Subsequent cleavage of the oxirane ring leads mainly to GDA-sa (**2**) along with small amounts of iso-GDA-sa (**3**). Goniodomic acid, has now been observed in reaction mixtures resulting from treatment of GDA with bases under anhydrous conditions. Goniodomic acid was shown to be formed by a pathway involving base-catalyzed attack of the C32 hydroxy group on C31, displacing the carboxyl group (Scheme 4). Formation of the resonance-stabilized carboxylate anion compensates for the strain introduced by the oxirane ring. Identification of CID fragment ions associated with the large chromatographic peak establishes GDA-sa as being the primary product of opening the macrolide ring. We and others have found evidence for base-catalysis of the conversion of GDA to GDA-sa, although there is significant disagreement as to observed rates of the cleavage reactions.^15,31,32^ Formation of oxirane intermediate **4** provides an explanation for the facile cleavage of the macrolide ring.

### 4.3 Opening the Oxirane Ring of Goniodomic Acid

**Scheme 5:**
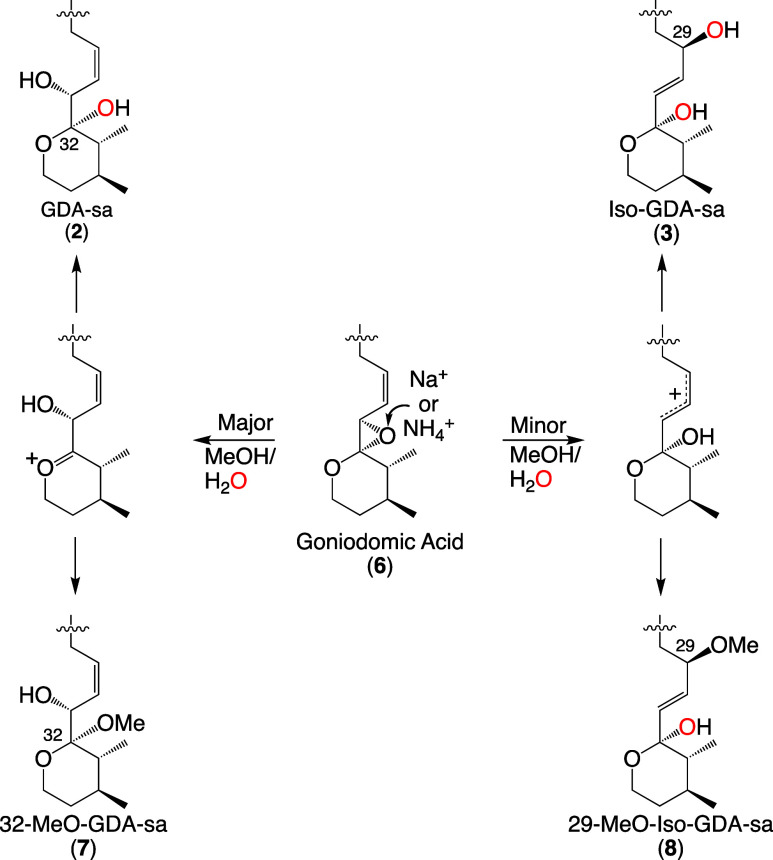
Two Pathways for Ring Opening by H_2_O and MeOH Upper sector: Hydrolytic ring-opening of goniodomic acid with 1:1 H_2_^18^O-MeOH gave mainly 32-^18^O GDA-sa (**2**), formed by attack at C32. A small amount of 29,32-^18^O_2_ iso-GDA-sa (**8**) was observed. The latter was formed by allylic attack at C29. Lower sector: Ring opening of goniodomic acid by MeOH gave mainly 32-MeO-GDA-sa (**7**). A small amount of 29-MeO-iso-GDA-sa (**8**) was observed. The latter was formed by allylic attack at C29. Red O = ^18^O.

The reaction of GDA with methanolic Na_2_CO_3_ gave goniodomic acid. Monosodio and disodio adducts were observed at *m*/*z* 791.3976 (C_43_H_60_NaO_12_^+^) and *m*/*z* 813.3799 (C_43_H_59_Na_2_O_12_^+^), respectively. Workup had involved removal of the MeOH by evaporation *in vacuo* followed by partitioning the residue between CH_2_Cl_2_ and H_2_O with the GD products being collected in the CH_2_Cl_2_ fraction. MS showed an additional carboxylic acid (Scheme 5, 32-MeO-GDA-sa (**7**), C_43_H_62_O_12_) had been formed (Table 7a). GDA-sa was present in the largest quantity, despite the reaction having been carried out under anhydrous conditions. GDA-sa must have been formed by hydrolysis of the oxirane ring during aqueous workup. Iso-GDA-sa was formed in small quantities. It could have arisen by allylic attack at C29 of goniodomic acid or on GDA itself. A small quantity of C29-methoxylated seco acid **7** was also observed, having arisen by a pathway analogous to that of iso-GDA-sa. Detection of the hydrolysis and methanolysis products and of goniodomic acid itself left little doubt that goniodomic acid had been formed in the reaction and was an intermediate in the formation of GDA-sa and iso-GDA-sa from GDA. The oxirane ring formed readily but was largely resistant to attack by methoxide during extended reaction with methanolic Na_2_CO_3_. This is consistent with the low reactivity of multiply substituted epoxides with nucleophiles. This sequence of events is strongly indicative of goniodomic acid having been formed during the reaction of GDA with methanolic Na_2_CO_3_.

Goniodomic acid had not been detected in our studies (Scheme 4) of the reactions carried out at pH 8 in 1:1 MeOH-H_2_O, but products and fragment ions arising from methanolysis reactions provided evidence that the oxirane compound had been present.^15^ Monosodio and disodio adducts of 32-methoxy-carboxylic acid (**7**, C_44_H_64_O_13_**)** were observed at *m*/*z* 823.4245 and 845.4085 (Table 3). Reaction carried out in 1:1 MeOH-H_2_^18^O gave these ions plus a small amount (<5%) of monosodio *m*/*z* 825.4291 bearing methoxy substitution and ^18^O label (C_44_H_64_O_12_^18^O). Due to formation of the disodio adduct, they can be assigned as adducts formed from goniodomic acid by methanol attack (Scheme 4, bottom). We hypothesize that the methoxy group is at C29 and the ^18^O label is at C32, i.e., 32-^18^O-**7** in Scheme 5, formed by allylic attack at C29 followed by ^16^O/^18^O exchange of the keto group of the ring-opened hemiketal.

The Na_2_CO_3_ reaction demonstrated that goniodomic acid has an extended lifetime if aqueous, acidic conditions are avoided. During workup of the reaction, ring-opening must have been acid-catalyzed even though the H^+^ concentration was less that 10^–11^ M. We believe that participation of Na^+^ as a Lewis acid catalyzed ring-opening (Scheme 4). More work will be needed to test this hypothesis but, in its support, mass spectra indicate that goniodomic acid actively coordinates with Na^+^, forming mono- and disodio adducts, *m*/*z* 791.3976 and 813.3799 (Table 7a). GDA-sa also formed complexes with Na^+^ but failed to form them with K^+^.^15^ GDA forms a weak complex with Na^+^ but strongly coordinates K^+^.^33^ In contrast to our observation of ring-cleavage of GDA by methanolic Na_2_CO_3_, Takeda reported that GDA failed to undergo ring-opening with methanolic K_2_CO_3_.^34^ This surprising difference is likely to be due to the K^+^ complex with GDA not involving the lactone carbonyl group.^8^

The reaction of GDA with methanolic NH_3_ gave similar results although one should keep in mind that the NH_3_ was ∼30-fold higher concentration than the Na_2_CO_3_. Little is known about the catalytic mechanism. NH_4_^+^ ion may be catalyzing ring-opening by proton donation to the oxirane oxygen atom. Alternatively, the NH_4_^+^ may form a polydentate complex with GDA which then promotes ring-opening. NMR and/or X-ray crystallographic studies need to be carried out on the NH_4_^+^ complex of GDA. CID fragmentation of the NH_4_^+^ adduct of GDA-sa is initiated by loss of NH_3_ plus a water molecule to yield protonated goniodomic acid, *m*/*z* 769. Sequential loss of four more water molecules yielded fragment ions at *m*/*z* 751, 733, 715, and 697 (Figure 4).

**Scheme 6:**
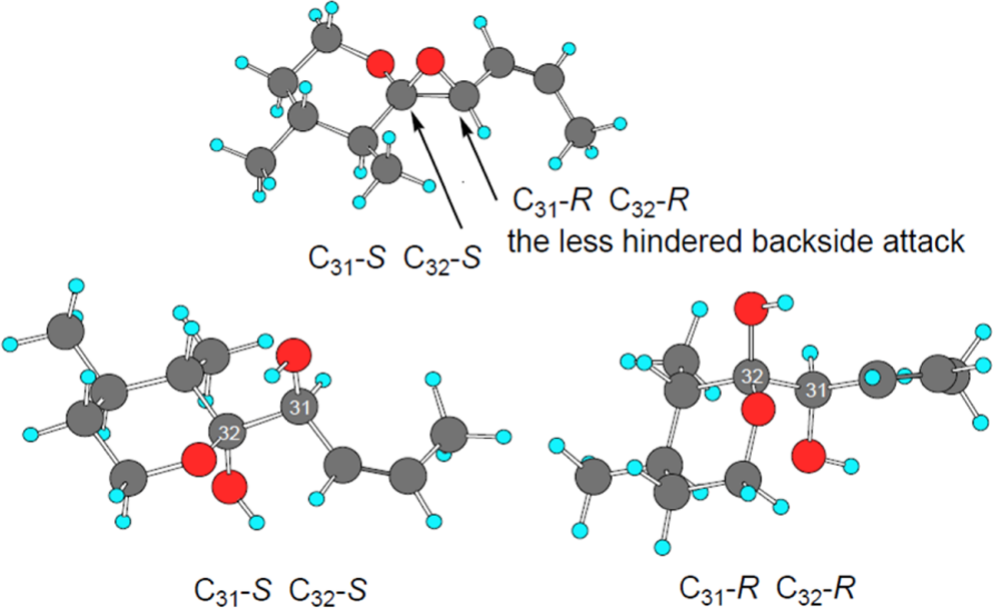
Diastereomeric Seco Acids Arising by Nucleophilic Attack on C31 and C32 of the Oxirane Ring of Goniodomic Acid^17^ Used with permission of the journal.

Hess and Smentek considered mechanisms by which ring-opening of goniodomic acid to form GDA-sa might occur.^17^ They concluded that under basic conditions ring-opening involved nucleophilic attack on the oxirane ring. They favored attack on C31 over C32, due to additional steric constraints for nucleophilic attack at the fully substituted C32 position (Scheme 6). It is our conclusion that under basic conditions, pH 8 and above, neither C31 nor C32 of goniodomic acid is a significant site for attack by nucleophiles. The ability of goniodomic acid to withstand extended treatment with methanolic Na_2_CO_3_ is strong evidence against GDA-sa being formed by direct nucleophilic attack on either C31 or C32 of the oxirane ring. Ring opening requires acid catalysis. With Na_2_CO_3_, the Na^+^ serves as a Lewis acid, accepting electrons from the oxirane oxygen atom. The dielectric constant (ε) of the reaction medium plays an important role in the ring-opening reaction. Ring opening will be much faster in water (ε 80) than in MeOH (ε 37).

### 4.4 Isomers of GD Seco Acids

**Scheme 7:**
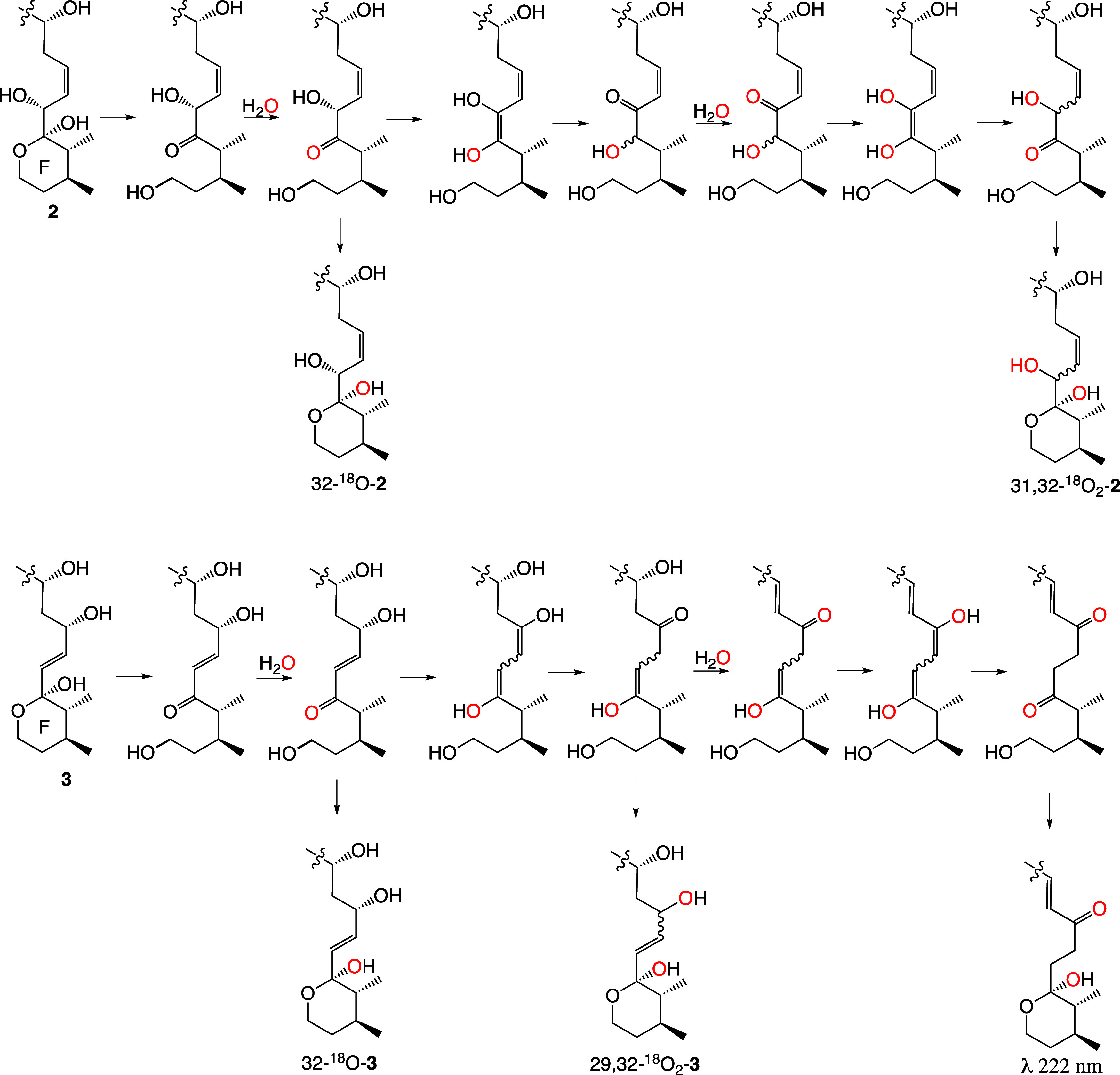
Isomers of GDA Seco Acids **2** and **3** ^18^O incorporation into the seco acids designated in red.

Goniodomic acid undergoes ring-opening mainly by reaction of H_2_O on C32 to give GDA-sa (**2**). A small amount of ring-opening by allylic attack at C29 yields iso-GDA-sa (**3**). When ring-opening takes place in H_2_^18^O, the allylic reaction places the ^18^O atom on C29. Tautomerization can transfer the carbonyl functionality from C32 to C29. This is followed by loss of H_2_O from C27 to form α,β-unsaturated ketone (bottom right of Scheme 7), accounting for the 222 nm UV spectrum. The 32-keto group can acquire ^18^O label by exchange and be in equilibrium with labeled hemiketal. Support is found in the CID spectrum where the RDA tail fragment contains the two ^18^O atoms (C_23_H_36_NaO_4_^18^O_2_^+^). Experimental evidence is not available for differentiating doubly labeled GDA-sa from its iso-GDA-sa counterpart.

In a related process, allylic methanolysis of goniodomic acid yielded the 29-methoxy analog **8** of iso-GDA-sa (Scheme 4). The ^18^O label could be acquired by exchange after the F ring has been opened to give ^18^O-labeled ketone which would reconvert to the hemiketal. Experimental support for this scenario can be found in the mass spectrum (Table 5) of the ^18^O-labeled 29-MeO-GDA-sa (**9**, *m*/*z* 825.4291, C_44_H_64_NaO_12_^18^O^+^). Seco acids **2** and **3** were both prone to loss of ^18^O-label during subsequent chromatography and other manipulation. The CID spectrum of the labeled GDA-sa arose in the same way as that of the unlabeled material but most (∼85%) of the ^18^O label was lost in the first dehydration step (Figure 5). The remainder of the ^18^O label was largely resistant during subsequent dehydration steps. H_2_^18^O was incorporated into both GDA-sa and iso-GDA-sa. The major site of labeling is the C32 hemiketal. It should be recognized that iso-GDA-sa could arise by allylic attack on C29 of goniodomic acid or of GDA itself although goniodomic acid would be expected to be the stronger electrophile. CID fragmentation of ^18^O-labeled **3** causes immediate loss of ^18^OH from C32, restoring the oxirane ring but without the ^18^O label. The ^18^O hydroxy group on C29 of **3** lacks the high reactivity of the C32 hydroxy group of GDA-sa and must compete with the other oxygen atoms during the remaining dehydration steps. The initial observations were made with the large 2.20 min chromatographic peak (Figure 5) but the observations also held true for the 2.30 min peak, as shown in Figure 7. The ratios of labeled to unlabeled dehydration products remain approximately the same throughout the dehydration steps.

GDA-sa (**2**) and iso-GDA-sa (**3**) undergo isomerizations initiated by opening of the hemiketal of ring F to yield a keto group at C32 (Scheme 6). The keto group becomes the basis for an array of isomerizations involving C27 through C32. The isomerizations include conversion of the configuration of the C29–C30 double bond from *Z* to *E*. The gradual shift of the UV spectrum from end absorption to a broad maximum at 222 nm is evidence that the carbonyl shift is occurring. The complexity of this array of structural and stereoisomers is compounded by iso-GDA-sa (**3**) undergoing similar transformations. Potential isomers of **2** and **3** are shown in Scheme 6. With **3**, an additional complication is the potential for loss of the β-hydroxy group on C27 once the keto group has shifted to C29.

The introduction of ^18^O during ring-opening of goniodomic acid in H_2_^18^O-MeOH provides support for the proposed equilibria. The incorporation of ^18^O at C31 and C32 of **2** is evidence for occurrence of enol-keto tautomerism between C31 and C32 of GDA-sa (Scheme 6). This suggests that epimerization at C31 is likely to have occurred along with conversion of the configuration of the double bond from *Z* to *E*. Similar isomers can be expected to arise with iso-GDA-sa. Loss of the C27 hydroxy group of iso-GDA-sa can occur when the carbonyl group is on C29. Collectively, a large number of isomers are created and to varying extents they are in equilibrium causing elucidation of structures of the individual isomers to be a daunting task.

Of note is the double labeling occurring during these transformations. The second label can be assigned as being on C31 where isotopic exchange had occurred during enol-keto tautomerism after opening of the hemiketal ring. As previously discussed, steric grounds preclude introduction of the isotopic label at C31 by direct attack of H_2_^18^O on C31 of GDA. Reversal of the reaction sequence then yields GDA-sa with ^18^O labels on both C31 and C32. This scenario is supported by the observation of precursor ions at *m*/*z* 813.4184 (C_43_H_62_NaO_11_^18^O_2_^+^) and 835.4004 (C_43_H_61_Na_2_O_11_^18^O_2_^+^) for mono- and disodio adducts, respectively. In reinforcement of these conclusions, double labeling was also observed in the CID spectrum of ^18^O-labeled GDA-sa. It showed the presence of the RDA tail fragment ion bearing single and double labels (*m*/*z* 433.2440, C_23_H_36_NaO_5_^18^O^+^ and *m*/*z* 435.2482, C_23_H_36_NaO_4_^18^O_2_^+^). These ions are again assigned to ^18^O-labeling of the C32-OH and C31-OH. Double labeling was not observed with iso-GDA-sa, perhaps due to the lower signal-to-noise.

For the seco acids, the presence of isomeric species limited the structural studies that could be undertaken. Most of the fragment ion masses were the same for the two chromatographic peaks but differences were readily observed for several of them. For signals observed for only one peak, the ones that were uniquely observed were invariably in the larger chromatographic peak, so caution should be exercised as to whether these are due to structural differences or only to the lower S/N of the smaller peak.

**Scheme 8:**
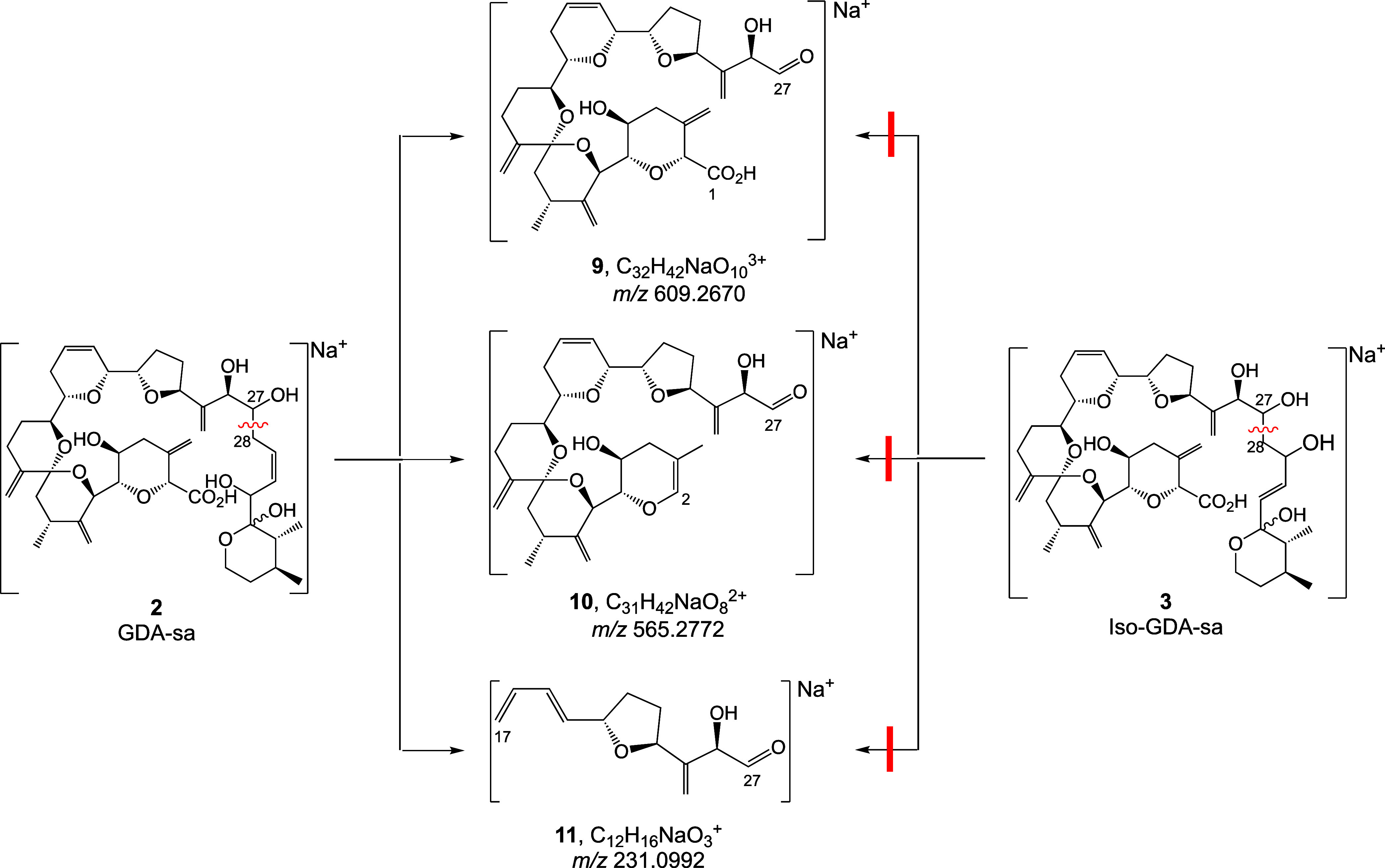
Grob–Wharton-Type CID Fragmentation between C27 and C28 of GDA-sa But Not of iso-GDA-sa Red lines indicate blocked fragmentations of **3**.

Signals for CID fragments at *m*/*z* 565, 495, and 231 were observed only in the larger chromatographic peak (Tables 4 and 5). Empirical formulas, were assigned from accurate mass measurements on spectra acquired by direct infusion on the FT-ICR spectrometer even though the signals were observed in composites of the two chromatographic peaks. The empirical formulas of the three ions are C_32_H_42_NaO_10_^+^, C_31_H_42_NaO_8_^+^, and C_12_H_16_NaO_3_^+^ and the structures of the three ions can be assigned as **9**–**11** (Scheme 8), arising by Grob–Wharton fragmentation of the C27–C28 bond. Iso-GDA-sa lacked the ability to carry out the 6-membered pericyclic process initiated by attack of the C27-OH on C30 of the double bond. It should be noted that iso-GDA-sa might seemingly be able to undergo Grob–Wharton cleavage by attack of the C27-OH on the distal end of the C25 double bond but this reaction is stereochemically suppressed by hydrogen-bonding between the vicinal C26 and C27 hydroxy groups. As a consequence, the major and minor chromatographic peaks are established to be GDA-sa (**2**) and iso-GDA-sa (**3**), respectively.

The *m*/*z* 395 ion was also only formed by the major chromatographic peak. It arose from RDA cleavage of the C16–C17 bond of GDA-sa, giving tail fragment *m*/*z* 431 followed by sequential loss of two H_2_O molecules. For iso-GDA-sa, the RDA cleavage occurred and loss of one H_2_O molecule but loss of a second H_2_O did not. Data are not available to identify the hydroxy group in **3** that was refractory to loss of the second H_2_O molecule.

**Scheme 9:**
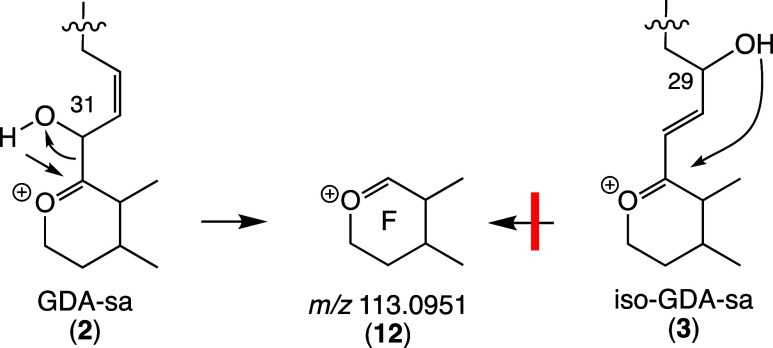
Formation of Oxenium Ion **12** by CID of GDA-sa But Not of iso-GDA-sa Red line indicates a prohibited fragmentation.

Differences were observed in the CID spectra of NH_4_^+^ adducts of the two chromatographic peaks (Figures 4–7). Usefulness of these ions was limited by poor S/N of the smaller peaks relative to spurious peaks. The most notable signal was an ion at *m*/*z* 113 that was seen with good S/N in the CID spectrum of the large peak but was not observed in the small one. It is assigned as **12** (C_7_H_13_O^+^), formed from ring F of GDA-sa by intramolecular proton transfer from the 31-OH to C32 of GDA-sa. The transfer involves a 4-membered ring (Scheme 9). An analogous process with iso-GDA-sa would involve a 6-membered ring. Usually, a 6-membered ring would be favored but in the present case the *E* configuration of the C30–C31 double bond sterically precludes intramolecular transfer of the proton from the C29 hydroxy group.

**Scheme 10:**
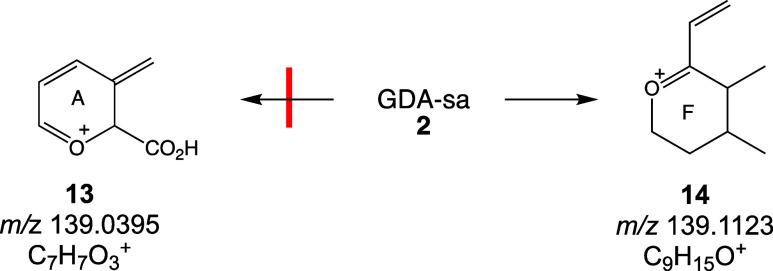
Potential Routes for Formation of *m/z* 139 Fragment Ions from Rings A and F of GDA-sa with the Latter (**14**) being More Likely

An *m*/*z* 139 ion was observed for both chromatographic peaks and for both the ^16^O and ^18^O samples (Figures 4–7). It has been used with transitions for quantitation of GDA, GDB, and GDC. The empirical formula might be either C_9_H_15_O^+^ or C_7_H_7_O_3_^+^. Potential structures are **13** and **14** (Scheme 10) derived from rings A and F, respectively. The *m*/*z* 139 ion was first observed by Sharma et al. in the electron impact spectrum of GDA.^35,36^ They assigned it as dihydrogeranyl cation (C_10_H_19_^+^) prior to the polyketide origin of GDA being known. More recently, the *m*/*z* 139 cation was observed in EI and ESI spectra of GDA.^16^ Hintze observed that 34-desmethyl-GDA yields an *m*/*z* 125 ion rather than 139. Hintze’s data suggest that the correct assignment for *m*/*z* 139 is **14** derived from ring F but exact mass measurement will be required to resolve this uncertainty.

### 4.5 Restoration of Goniodomic Acid during CID of GDA-sa

**Scheme 11:**
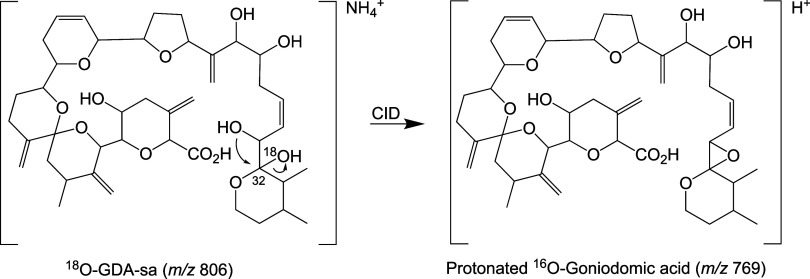
Reversion of GDA-sa to Goniodomic Acid

Conversion of goniodomic acid to GDA-sa was found to be a reversible process during CID fragmentation of GDA-sa. The reverse reaction was observed with the NH_4_^+^ adduct of GDA-sa (Scheme 11). With ^18^O-labeled GDA-sa (Figure 5), loss of NH_3_ and labeled H_2_O gave unlabeled protonated goniodomic acid **(6**, *m*/*z* 769), which then underwent a cascade of additional dehydration steps. This observation is attributed to the ^18^OH being the hemiketal hydroxy group. It is instructive to recognize that due to involvement of the oxirane intermediate not only is C32 the preferred site of introduction of the hydroxy group in the reaction of GDA with water but the C32 hydroxy group is also the most vulnerable to loss during CID where loss of ^18^O involved reversal of the process by which the seco acid had been formed. Na^+^ adducts are much less prone to loss of ^18^O. ^18^O-labeled ions were observed at *m*/*z* 793, 767, 749, 731, 697, 433, and 415 for the monosodio *m*/*z* 811 precursor ion and at *m*/*z* 435, 433, 425, and 415 for the disodio *m*/*z* 833 precursor ion.

The ^18^OH introduced by allylic attack at C29 during formation of iso-GDA-sa lacks the special properties of the C32 hydroxy group of GDA-sa and is no more vulnerable to dehydration than hydroxy groups at other sites. As a consequence, the ratio of labeled to unlabeled hydroxy groups at *m*/*z* 771/769, 753/751, 735/733, 717/715, and 699/697 in the CID spectrum of NH_4_^+^ adducts remains essentially constant as five successive water molecules are lost from GDA-sa (Figures 5 and 7). The C32 selectivity for dehydration is not as apparent with the sodio adduct of GDA-sa where dehydration is overshadowed by decarboxylation of ring A and RDA fragmentation of ring D.

### 4.6 Precedents for the Goniodomic Acid Chemistry

**Scheme 12:**
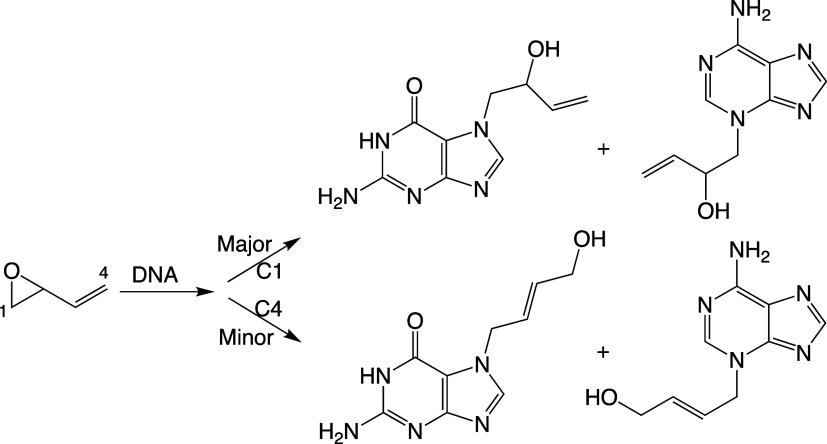
Main Adducted Nucleobases Resulting from Reactions of the Epoxide of Butadiene with DNA Arise by Direct Attack at C1 rather than Allylic Attack at C4

The chemistry of butadiene monoxide provides a line of evidence that argues against allylic attack being a major pathway for ring-opening of goniodomic acid. The epoxides of butadiene have been studied extensively due to butadiene being a high-volume industrial chemical and the epoxides being mutagenic and carcinogenic due to their reactions with DNA. The DNA adducts of 1,2-epoxy-3-butene are dominated by attack of guanine and adenine on C1 with lesser amounts of allylic attack at C4 as shown in Scheme 12, reflecting the steric requirements that must be met to achieve allylic attack.^37−39^

**Scheme 13:**
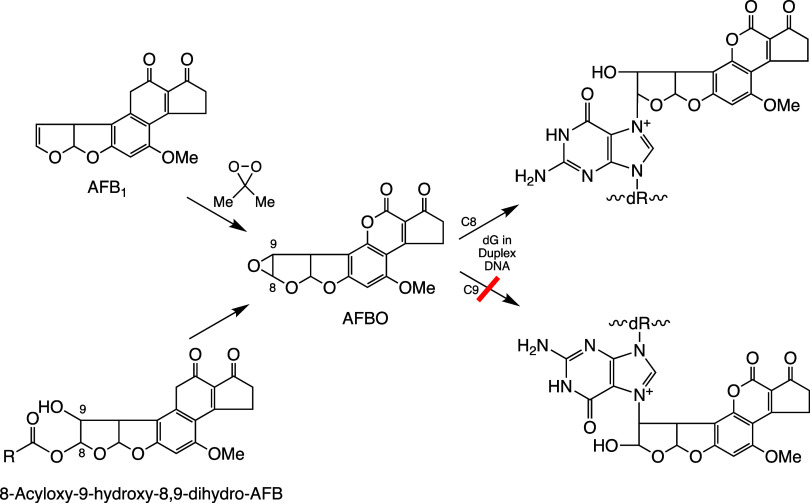
Chemistry of Aflatoxin Preparation of AFBO by reaction of AFB1 with dimethyldioxirane under aprotic conditions (upper left). Alternative formation of AFBO from 8-acyloxy-9-hydroxy-8,9-dihydro-AFB (lower left). Reaction of AFB1 with 8-acyloxy-9-hydroxy-8,9-dihydro-AFB with dG of DNA by first forming AFBO. Reaction of AFBO with deoxyguanosine occurs exclusively by cleavage of the bond between C8 of AFBO and the oxirane oxygen atom (upper right). Reaction is not observed at C9 of AFBO (lower right).

Oxiranes vary widely in their reactivity. For example, ethylene oxide is highly susceptible to nucleophilic attack but oxiranes bearing substituents become resistant to attack due to steric hindrance. On the other hand, electron-donating substituents on the ring can make oxiranes hyperreactive, vulnerable to acid-catalyzed attack, even at pH values of 7 and above. A notable example is the 8,9-epoxide (AFBO) of aflatoxin B_1_ (AFB1) shown in Scheme 13. AFB1 is a potent mutagen. The epoxide is, in fact, its activated form.^40^*In vivo*, this trisubstituted oxirane ring fused to the tetrahydrofuran ring has a fleeting existence due to ring-opening.^41^ The epoxide can be synthesized by reaction of AFB1 with dimethyldioxirane in aprotic media.^42^ Opening of the oxirane ring of AFBO by deoxyguanosine in DNA and by other nucleophiles occurs exclusively by cleavage of the ketal C–O bond, equivalent to that of the C32–O bond in goniodomic acid. The AFBO reaction is acid-catalyzed, even under mildly basic conditions. Acid-catalysis is likely to be a combination of Brønsted and Lewis catalysis. The reaction occurs exclusively at C8 of AFBO. The rationale for this selectivity is that positive charge on C8 in the transition state is stabilized by donation of electron density from the adjacent tetrahydrofuran oxygen atom.^43^ 8-Acyloxy-9-hydroxy-8,9-dihydro-AFB also reacts with dG of DNA by first forming AFBO. The presence of the 9-OH is essential for forming AFBO and thereby the adducts of AFBO.^44^ The reaction of AFBO with deoxyguanosine occurs exclusively by cleavage of the bond between C8 of AFBO and the oxirane oxygen atom. This is directly comparable to the situation with goniodomic acid.

Another prominent example of a sensitive epoxide is leukotriene A4 (LTA4) in which the epoxide ring is exquisitely sensitive to acid-catalyzed solvolysis. LTA4 long eluded isolation but Borgeat and Samuelsson were able to obtain evidence for its existence via trapping experiments.^45^ Subsequently, the methyl ester of LTA4 was found to be stable enough to be isolated when protected from acidic conditions.^46^ The methyl ester could then be converted to the Na^+^ salt by treatment with NaOH.^47^

### 4.7 Potential Biological Consequences

**Figure 8:**
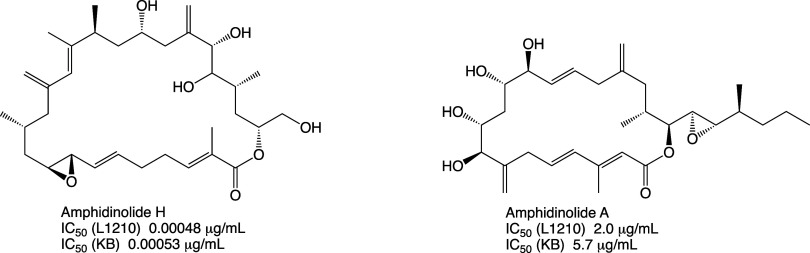
Amphidinolides A and H.

Extensive toxicological studies of the effects of GDA on actin have revealed complex modes of action resulting from stabilization of actin filaments^9−14^ but similar studies have not yet been carried out on newly discovered congeners. Of note is goniodomic acid, the subject of this paper. Goniodomic acid has the ring F oxane moiety linked to the oxirane ring, enhancing the susceptibility of the oxirane ring to acid-catalyzed attack by nucleophiles. The C29–C30 double bond creates a second route for attack by nucleophiles. These relationships may increase the reactivity of goniodomic acid with actin relative to that of GDA. Structural parallels exist for goniodomic acid with certain of the amphidinolides produced by dinoflagellate species of the genus *Amphidinium*. Kobayashi and co-workers observed enhanced toxicity toward murine L1210 and human KB cell lines by amphidinolide H and other amphidinolides that bear a vinyloxirane moiety (e.g., AmpB1, AmpB4, AmpN, etc.) relative to many others that lack this structural feature (e.g., amphidinolide A).^48,49^ The toxicities of these activated amphidinolides are enhanced by more than 4 orders of magnitude (Figure 8). Usui et al. found evidence of the enhanced activity of AmpH being due to covalent binding to actin.^50^ Study of the interaction of goniodomic acid with actin is recommended to discover whether goniodomic acid might be the principal toxin in the goniodomin group.

## 5 Conclusions

Our new understanding of the process by which GDA-sa is formed involves base-catalyzed attack of the hemiketal hydroxy group of GDA on C31, displacing the carboxylate anion to create goniodomic acid (**4**) which contains an oxirane ring. Resonance stabilization of the carboxylate ion makes the reaction thermodynamically favored in mild base despite strain being induced by the oxirane ring. The oxirane ring is inherently unstable, undergoing facile ring-opening by solvolytic cleavage of the C32–O bond to give GDA-sa (**2**). The ring-opening reaction is acid-catalyzed, probably by Na^+^. The large, fast-eluting chromatographic peak observed in Figure 3 is assigned as GDA-sa (**2**). In H_2_^18^O media, the primary reaction introduces the ^18^O label into GDA-sa at C32. Reversal is observed in the mass spectrometer. The preferred axial orientation of the C32 hydroxy group of **2** provides a clear pathway for backside displacement by the 31-OH, leading to restoration of the oxirane ring but with loss of the ^18^O label. The smaller, more slowly eluting chromatographic peak is assigned as C29-substituted iso-GDA-sa (**3**) which arises by allylic attack on goniodomic acid and/or by allylic attack on GDA. In either case, ^18^O is introduced at C29. Allylic attack is a minor pathway.

## Supporting Information Available

Products of the reaction of GDA with 1:1 MeOH-H_2_O, pH 8.0 sodium phosphate, Na^+^ adduct (Figure S1); products of GDA in 1:1 MeOH/H_2_^18^O, pH 8.0 sodium phosphate, Na^+^ adducts (Figure S2); CID fragmentation of GDA-sa, precursor ion *m/*z 831, Na^+^ adduct, GDA-sa formed by sodium phosphate, pH 8.0 in 1:1 MeOH-H_2_O (Figure S3); CID fragmentation of ^18^O-GDA-sa, precursor ion *m*/*z* 833, Na^+^ adduct; GDA-sa formed by sodium phosphate, pH 8.0 in 1:1 MeOH-H_2_^18^O (Figure S4); reaction of GDA with anhydrous, methanolic Na_2_CO_3_, aqueous workup, Na^+^ adduct (Figure S5); polar product ions from reaction of GDA with anhydrous, methanolic ammonia, Na^+^ adducts (Figure S6); methyl ester of GDA-sa, Na^+^ adduct, *m*/*z* 823.4236, prepared with anhydrous, methanolic NH_3_ (Figure S7); CID fragmentation of the methyl ester of GDA-sa, *m*/*z* 823.4236, formed by reaction of GDA with anhydrous, methanolic NH_3_, Na^+^ adduct (Figure S8) (PDF)
