# Supplementary material for: Goniodomic Acid, a Transient Oxirane Intermediate in the Conversion of the Macrolide Algal Toxin Goniodomin A to Seco Acids
Source: Chem Res Toxicol. 2024 Dec 23;38(1):163–81. doi: 10.1021/acs.chemrestox.4c00390 (PMC11752514; doi:10.1021/acs.chemrestox.4c00390)
Supplement: Supplementary file 1 — tx4c00390_si_001.pdf [file tx4c00390_si_001.pdf]

Supporting Information

Goniodomic acid, a transient oxirane intermediate in the conversion of the macrolide algal toxin goniodomin A to seco acids

Constance M. Harris,[a] Bernd Krock,[b] and Thomas M. Harris (thomas.m.harris@vanderbilt.edu)[a]*

[a] Department of Chemistry, Vanderbilt University, Nashville, Tennessee 37235, USA
[b] Alfred-Wegener-Institut Helmholtz-Zentrum für Polar- und Meeresforschung (AWI), 27570 Bremerhaven, Germany

Supplemental Figures

Figure S1. Products of the reaction of GDA with 1:1 MeOH-H_2O, pH 8.0 sodium phosphate. Na^+ adduct. See Table 3a.

Figure S2. Products of GDA in 1:1 MeOH/$H_2{}^{18}O$, pH 8.0 sodium phosphate. Na^+ adducts. See Table 3b.

Figure S3. CID fragmentation of GDA-sa, precursor ion *m/*z 831, Na^+ adduct. GDA-sa formed by sodium phosphate, pH 8.0 in 1:1 MeOH-H_2O. See Table 4.

Figure S4. CID fragmentation of ^{18}O-GDA-sa, precursor ion *m/z* 833, Na^+ adduct. GDA-sa formed by sodium phosphate, pH 8.0 in 1:1 MeOH-$H_2{}^{18}O$. See Table 5.

Figure S5. Reaction of GDA with anhydrous, methanolic Na_2CO_3, aqueous work-up, Na^+ adduct. See Table 7a.

Figure S6. Polar product ions from reaction of GDA with anhydrous, methanolic ammonia, Na^+ adducts. See Table 7c.

Figure S7. Methyl ester of GDA-sa, Na^+ adduct, *m/z* 823.4236, prepared with anhydrous, methanolic NH_3. See Table 7c.

Figure S8. CID fragmentation of the methyl ester of GDA-sa, *m/z* 823.4236, formed by reaction of GDA with anhydrous, methanolic NH_3, Na^+ adduct. See Table 7c.

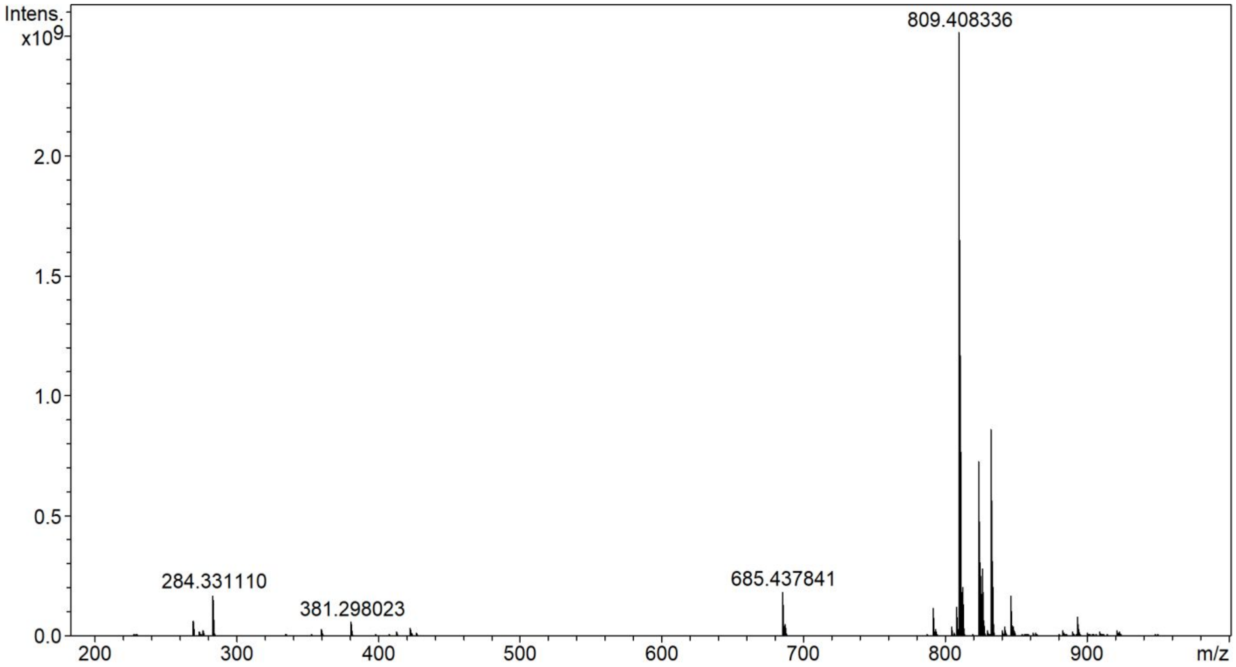

Figure S1. Products of the reaction of GDA with 1:1 MeOH-H_2O, pH 8.0 sodium phosphate. Na^+ adduct. See Table 3a.

#	Observed, *m/z*	Intensity	Formula	Calc'd, *m/z*	Error, ppm
382	863.418048	4747055			
381	863.403303	1729838			
380	863.329424	10944302			
379	863.314342	4260655			
378	862.385411	3600175			
377	862.369758	1408815			
376	861.382518	9381679			
375	861.366341	3437359			
374	858.764933	2226994			
373	857.761882	3925812			
372	857.752492	1720628			
371	856.749203	6255413			
370	856.731485	2070837			
369	856.457841	2051125			
368	856.369429	2507574			
367	855.744085	8880950			
366	855.728207	3517238			
365	855.452548	3177271			
364	855.437030	1282871			
363	855.363487	4638519			
362	855.348059	1946935			
361	854.730786	3097399			
360	854.715791	1425207			
359	853.726868	6821688			

358	853.712875	2316088			
357	852.714732	1235768			
356	851.711470	1490743			
355	849.375412	2413875			
354	849.365454	1554227			
353	848.421275	1269551			
352	848.371687	14413615			
351	848.354435	3142447			
350	847.417298	6932267			
349	847.399682	1469739			
348	847.368230	39853868			
347	847.351148	6151979			
346	846.504833	1339174			
345	846.413042	44097316			
344	846.396153	6389541			
343	845.408512	174350112	$C_{44}H_{63}Na_2O_{13}^+$	845.4059	3.14
342	845.397851	1974046			
341	845.392491	11923230			
340	845.346572	1197854			
339	844.351185	1424149			
338	843.442858	1303820			
337	843.353796	2611979			
336	843.347428	3059467			
335	843.339900	1133323			
334	842.439248	4676353			
333	842.425230	1372160			
332	842.350472	12562175			
331	842.336558	2451199			
330	841.727192	1732855			
329	841.436135	11351795			
328	841.421618	2427635			
327	841.409258	1722611			
326	841.406211	1445619			
325	841.347579	37953264			
324	841.332715	4535026			
323	840.426427	1119460			
322	840.403632	7458532			
321	840.388449	1566435			
320	840.335965	1460963			
319	839.712963	1341144			
318	839.423912	2427603			
317	839.401437	21450450			
316	839.392201	1936082			
315	839.384968	2952914			
314	839.354674	1485778			
313	839.333829	3413201			
312	834.402072	3524188			
311	833.513614	1485888			
310	833.398137	24542780			
309	833.391486	3498556			
308	833.384455	2895420			
307	832.450650	2700828			
306	832.414109	1184283			

305	832.394638	318725664			
304	832.388049	6508058			
303	832.386172	5330458			
302	832.384504	5359130			
301	832.380752	9457178			
300	831.419231	1214966			
299	831.417463	1356278			
298	831.415716	1468918			
297	831.413884	1850870			
296	831.412103	1722870			
295	831.410294	2315766			
294	831.408535	2663926			
293	831.406700	3431926			
292	831.404924	4201974			
291	831.403121	5410294			
290	831.401374	7503350			
289	831.399551	10362358			
288	831.397792	16014837			
287	831.396006	27811318			
286	831.394065	70147576			
285	831.390712	859659776	$C_{43}H_{61}Na_2O_{13}^+$	831.3902	0.61
284	831.383724	9596405			
283	831.377098	18546164			
282	831.358389	1276404			
281	830.733175	1678299			
280	830.382048	7595469			
279	830.334716	1790923			
278	829.729959	3396018			
277	829.378382	23149986			
276	829.361992	1553313			
275	828.717943	3338628			
274	828.426855	1126518			
273	828.394307	1570677			
272	828.388433	2321013			
271	827.713892	5023572			
270	827.422998	1811781			
269	827.391688	7181636			
268	827.383415	4556099			
267	827.376998	1648451			
266	826.701922	1338655			
265	826.438624	2504977			
264	826.435611	2849041			
263	826.388071	65281296			
262	826.377918	2524942			
261	826.373156	2391822			
260	825.695746	2325736			
259	825.431976	23182552			
258	825.418061	1844440			
257	825.407630	2128088			
256	825.397459	1575127			
255	825.393710	2663639			
254	825.391811	3544279			
253	825.390002	5223639			

252	825.388226	9534678			
251	825.383498	283925728			
250	825.374936	5485782			
249	825.369819	6180053			
248	824.440329	1993886			
247	824.436650	3648670			
246	824.434930	5885086			
245	824.433154	10173597			
244	824.428340	311909536			
243	824.420307	5567645			
242	824.414741	5770396			
241	824.373796	2722458			
240	824.342508	3522712			
239	824.339700	3674264			
238	823.443573	1996895			
237	823.441695	2247775			
236	823.439851	2847838			
235	823.438225	3667038			
234	823.436422	4635742			
233	823.434654	6089822			
232	823.432883	8518750			
231	823.431139	13376606			
230	823.429336	23010398			
229	823.427405	61398108			
228	823.424455	734092352	$C_{44}H_{64}NaO_{13}^{+}$	823.4239	0.66
227	823.414753	5975133			
226	823.410989	10357852			
225	823.370764	5499482			
224	823.368798	5829722			
223	823.337418	8373336			
222	819.476433	3322685			
221	818.471518	5951721			
220	813.423501	1827071			
219	812.423240	7518351			
218	812.419759	12714126			
217	812.411065	1519246			
216	811.416763	204429344			
215	811.407018	5889049			
214	810.442865	1235875			
213	810.441138	1412003			
212	810.439410	1499043			
211	810.437740	1518498			
210	810.436026	2066338			
209	810.434280	2201506			
208	810.432626	2555810			
207	810.430931	3045282			
206	810.429222	3635105			
205	810.427500	4425633			
204	810.425819	5494689			
203	810.424110	7034785			
202	810.422406	9295776			
201	810.420720	13539232			
200	810.419025	19912608			

199	810.417319	34232224			
198	810.415476	89741216			
197	810.412265	1171871616			
196	810.405815	14219166			
195	810.400946	7784350			
194	810.397342	10986397			
193	810.395349	10499997			
192	810.386801	2602908			
191	810.385182	2443164			
190	810.381658	1664923			
189	810.379890	1496987			
188	809.664312	1512769			
187	809.455866	1176358			
186	809.454039	1140518			
185	809.450749	1434405			
184	809.448951	1270565			
183	809.447359	1500965			
182	809.445545	1646372			
181	809.443926	1982244			
180	809.442209	1809188			
179	809.440480	2303780			
178	809.438868	2418468			
177	809.437082	2598691			
176	809.435434	3094307			
175	809.433718	3540771			
174	809.432037	3761955			
173	809.430336	4474659			
172	809.428614	5113634			
171	809.426940	6207266			
170	809.425247	7182114			
169	809.423534	8804130			
168	809.421836	11261729			
167	809.420145	14817057			
166	809.418455	19715872			
165.	809.416766	27744032			
164.	809.415071	42817312			
163.	809.413353	77158176			
162.	809.411318	212555552			
161	809.408336	2519815936	$C_{43}H_{62}NaO_{13}^+$	809.4083	0.09
160	809.401792	44439328			
159	809.400094	25073438			
158	809.397895	13244190			
157	809.396377	13053726			
156	809.392989	15148830			
155	809.391348	16484125			
154	809.388181	8824605			
153	809.386374	6567709			
152	809.384614	5601053			
151	809.383064	3895068			
150	809.381134	3561244			
149	809.379469	3505948			
148	809.377823	2846492			
147	809.376111	2746139			

146	809.374501	2171675			
145	809.372668	1795867			
144	809.370951	1815323			
143	809.369305	1488667			
142	809.367586	1654554			
141	809.364219	1370906			
140	809.362484	1284890			
139	808.406546	2182811			
138	808.397887	27244186			
137	808.388384	3077785			
136	808.379950	2058903			
135	808.376718	1460375			
134	807.409362	1141266			
133	807.394907	123360784			
132	807.388344	3448335			
131	807.384675	4388367			
130	807.376782	4462094			
129	806.464148	2562444			
128	805.459985	13939960			
127	804.455982	38515808			
126	803.442496	1126851			
125	802.441032	2404132			
124	801.695898	1218218			
123	799.681324	1720409			
122	793.408517	4028662			
121	792.609436	1301856			
120	792.404336	29404218			
119	792.398414	2857017			
118	792.392963	1135928			
117	791.408402	2359166			
116	791.405009	5340030			
115	791.403362	10799997			
114	791.400450	116771712	$C_{43}H_{60}NaO_{12}^{+}$	791.3977	3.48
113	791.390741	2707323			
112	791.385079	1463802			
111	787.449334	2665098			
110	786.445557	7864265			

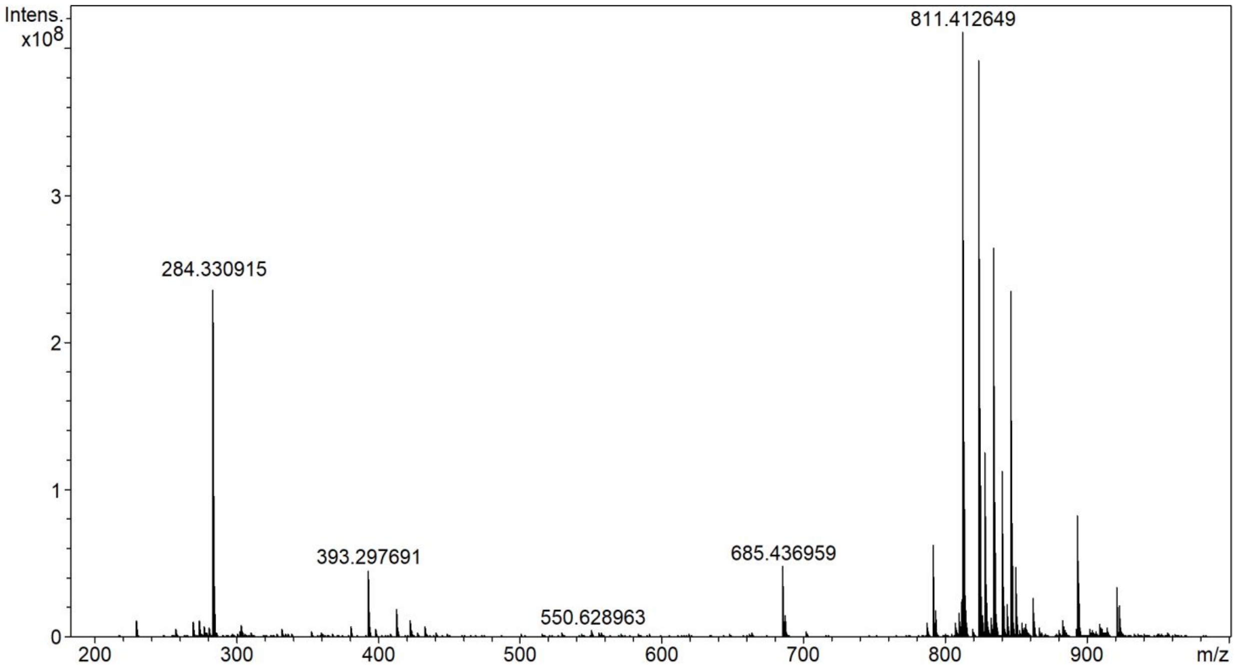

Figure S2. Products of GDA in 1:1 MeOH/$H_2{}^{18}O$, pH 8.0 sodium phosphate. Na^+ adducts. See Table 3b.

#	Observed, *m/z*	Intensity	Formula	Calc'd, *m/z*	Error, ppm
379	863.386853	1993771			
378	863.377764	1181739			
377	863.328072	907304			
376	862.384226	10025469			
375	861.381104	26737102			
374	858.763451	1512524			
373	858.368141	822072			
372	857.759927	2956569			
371	857.750489	1539864			
370	857.363930	1570052			
369	856.746521	4660963			
368	856.455934	736468			
367	856.367012	2877135			
366	855.742521	8986797			
365	855.732451	966829			
364	855.451384	1725597			
363	855.362208	6291608			
362	854.729601	4497525			
361	853.726046	9732157			
360	852.713953	1635842			
359	852.376194	1689327			
358	852.369569	772590			
357	851.710328	3305927			
356	851.375323	3869619			
355	851.372957	4334515			

354	851.366152	1555891			
353	850.372875	14014327			
352	850.359141	755318			
351	849.370226	48273208			
350	849.356916	1452344			
349	848.506427	1221891			
348	848.419012	1351677			
347	848.415067	1572093			
346	848.370601	2141946			
345	847.415277	8935101			
344	847.366939	4330170			
343	847.349175	858105			
342	846.411411	77911672			
341	846.360462	1395577			
340	846.355439	1306232			
339	845.416130	3015226			
338	845.412341	7312954			
337	845.410569	14898746			
336	845.406933	237459008	$C_{44}H_{63}Na_2O_{13}^+$	845.4059	1.23
335	845.401551	8447545			
334	845.399812	8107577			
333	845.398110	6018617			
332	845.357663	2460726			
331	845.350194	1852214			
330	844.745287	993037			
329	844.443081	1209336			
328	844.354086	7253490			
327	843.741789	1608136			
326	843.685918	1055236			
325	843.439035	2753459			
324	843.428826	953779			
323	843.350469	22664622			
322	843.338641	2814893			
321	842.729019	2306946			
320	842.405351	1524075			
319	842.398394	1032043			
318	842.349252	1168999			
317	841.726103	4638011			
316	841.435649	1070566			
315	841.404985	5282084			
314	841.396172	2547492			
313	841.354906	955937			
312	841.346189	2010912			
311	840.714253	1695731			
310	840.402504	33903836			
309	839.711785	3422889			
308	839.399393	113366160			
307	839.394256	6147218			
306	839.390294	3384466			
305	837.697070	1374995			
304	837.406021	1272061			
303	836.404118	6160304			
302	835.400377	15350625	$C_{43}H_{61}Na_2O_{11}{}^{18}O_2^+$	835.3997	0.84

301	835.386656	1154400			
300	834.398500	95376144			
299	834.386929	4784913			
298	833.415599	871619			
297	833.410168	1180354			
296	833.408318	1391298			
295	833.404714	2328258			
294	833.402893	3111618			
293	833.401033	4887234			
292	833.394305	269374144	$C_{43}H_{61}Na_2O_{12}{}^{18}O^+$	833.3945	-0.18
291	833.387060	8411840			
290	833.383507	8972992			
289	833.376870	2584256			
288	832.394184	5549680			
287	832.382312	1056622			
286	831.401620	754205			
285	831.391002	13345308	$C_{43}H_{61}Na_2O_{13}{}^+$	831.3902	0.95
284	830.730767	1644517			
283	830.395810	4098505			
282	830.387903	1438664			
281	829.727513	3048336			
280	829.425959	1033846			
279	829.392817	10213747			
278	829.386399	2877811			
277	829.383371	2087283			
276	829.374037	775794			
275	828.715593	2962233			
274	828.391853	36360476			
273	828.380527	4051228			
272	827.712244	4817122			
271	827.436720	895434			
270	827.387688	125849800			
269	827.377115	8296645			
268	826.699391	1679753			
267	826.432865	3972209			
266	826.386921	3053165			
265	825.694941	2975791			
264	825.431290	14917655			
263	825.429070	12403223	$C_{44}H_{64}NaO_{12}{}^{18}O^+$	825.4282	1.14
262	825.414119	941589			
261	825.383065	6859795			
260	825.370914	1624594			
259	825.343455	798735			
258	824.441399	965565			
257	824.437765	1847229			
256	824.434145	3332028			
255	824.432385	5599164			
254	824.430717	11177916			
253	824.427306	155733952			
252	824.418609	4006843			
251	824.339768	3297204			
250	823.679364	763127			
249	823.446298	941410			

248	823.439082	1629025			
247	823.437366	2025313			
246	823.435501	2452321			
245	823.433796	3467104			
244	823.431980	4841312			
243	823.430214	7372640			
242	823.428447	12947296			
241	823.426550	30789472			
240	823.423597	397234016	$C_{44}H_{64}NaO_{13}^{+}$	823.4239	-0.38
239	823.415274	5136223			
238	823.413677	5728095			
237	823.335935	7574359			
236	820.474676	1306441			
235	819.474008	2884585			
234	818.469882	5671303			
233	815.709654	931187			
232	815.424523	1835094			
231	814.697838	816908			
230	814.421089	9450479			
229	813.696154	1691044			
228	813.418446	28248968	$C_{43}H_{62}NaO_{11}{}^{18}O_{2}$	813.4168	2.03
227	813.406178	2377094			
226	812.417137	134368032			
225	812.405958	9655070			
224	811.680400	914897			
223	811.440092	794807			
222	811.429668	1761974			
221	811.428055	2041526			
220	811.426202	2365110			
219	811.424563	3285686			
218	811.422819	4065974			
217	811.421171	5515958			
216	811.419413	8456885			
215	811.417723	14084789			
214	811.416075	28953268			
213	811.412649	412175040	$C_{43}H_{62}NaO_{12}{}^{18}O^{+}$	811.4125	0.11
212	811.407816	11987636			
211	811.402995	24685236			
210	811.397480	5554867			
209	811.392269	2847923			
208	810.412473	6974026			
207	810.401209	1660744			
206	809.408836	16701917	$C_{43}H_{62}NaO_{13}^{+}$	809.4083	0.66
205	809.397492	3317212			
204	808.461270	1213814			
203	808.375737	2138476			
202	807.461747	3770120			
201	807.372973	4757758			
200	806.458818	9728152			
199	806.446966	1787031			
198	804.454357	1606071			
197	801.695135	1323644			
196	800.683237	881672			

195	799.681021	1870228			
194	797.665231	1418922			
193	793.405640	2474682			
192	792.403006	17783366			
191	791.399558	62423504	$C_{43}H_{60}NaO_{12}^{+}$	791.3977	2.28
190	788.449248	760189			
189	787.679291	1071781			
188	787.446827	3405834			
187	786.667790	890034			
186	786.444293	9267096			
185	786.322633	824075			
184	785.665039	1430336			

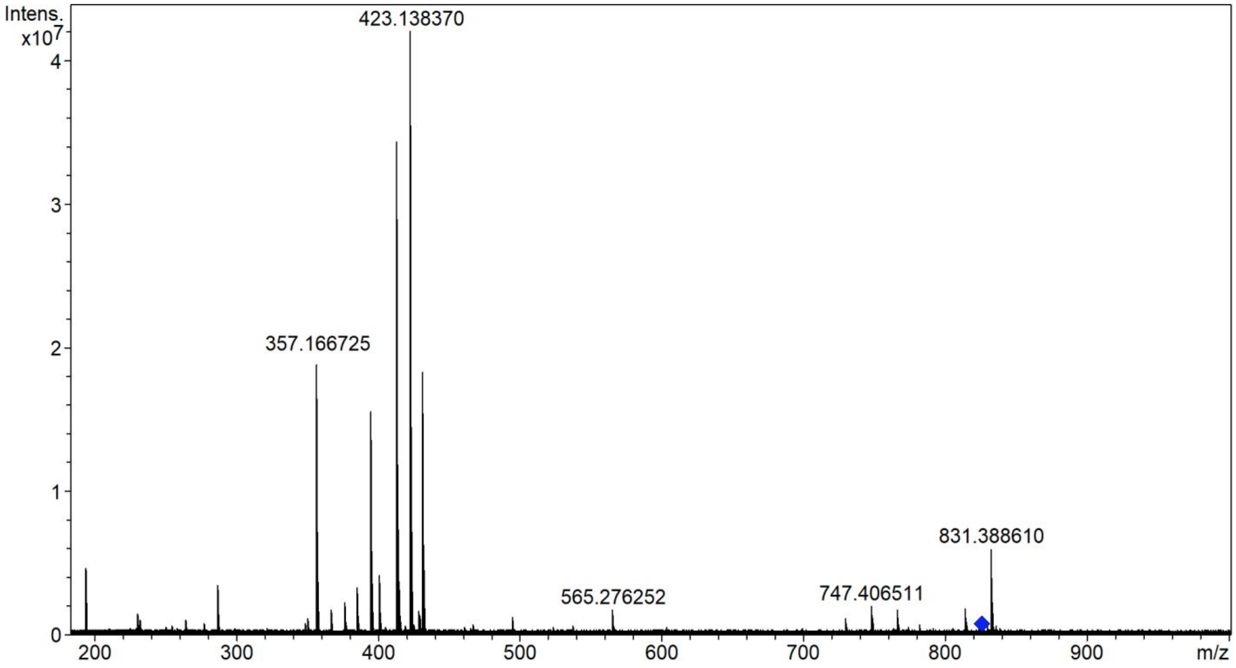

Figure S3. CID fragmentation of GDA-sa, precursor ion *m/z* 831, Na^+ adduct. GDA-sa formed by sodium phosphate, pH 8.0 in 1:1 MeOH-H_2O. See Table 4.

#	Observed, *m/z*	Intensity	Forrmula	Calc'd, *m/z*	Error, ppm
77	835.492498	686795			
76	833.395326	603302			
75	832.391831	2262165			
74	831.388610	6049923	$C_{43}H_{61}Na_2O_{13}^+$	831.3902	-0.66
73	829.373664	624224			
72	823.422453	740350			
71	814.381693	900225			
70	813.378579	1889141	$C_{43}H_{59}Na_2O_{12}^+$	813.3796	-1.28
69	791.395719	496266	$C_{43}H_{60}NaO_{12}^+$	791.3977	-2.52
68	781.391205	693004			
67	773.385908	580620	$C_{43}H_{58}NaO_{11}^+$	773.3871	-1.59
66	766.420457	690430			
65	765.417198	1781499	$C_{42}H_{62}NaO_{11}^+$	765.4184	-1.61
64	763.380909	468210			
63	748.409691	1138789			
62	747.406511	2034774	$C_{42}H_{60}NaO_{10}^+$	747.4079	-1.83
61	730.399701	532090			
60	729.396081	1182176	$C_{42}H_{58}NaO_9^+$	729.3973	-1.65
59	698.596464	491142			
58	603.177191	548996			
57	566.279609	663856			
56	565.276252	1750685	$C_{31}H_{42}NaO_8^+$	565.2772	-1.57
55	565.102184	483034			
54	537.281417	597738	$C_{30}H_{42}NaO_7^+$	537.2823	-1.63
53	523.944076	514375			
52	523.911222	480901			
51	495.234531	1197436	$C_{27}H_{36}NaO_7^+$	495.2353	-1.66

50	467.239746	716206	$C_{26}H_{36}NaO_6^+$	467.2404	-1.52
47	433.246454	591345			
46	433.243985	561137			
45	432.243097	5413750			
44	431.239770	18446200	$C_{23}H_{36}NaO_6^+$	431.2404	-1.41
43	430.227465	1335417			
42	429.224141	1639542	$C_{23}H_{34}NaO_6^+$	429.2248	-1.54
41	425.286779	646871			
40	425.145131	723669			
39	424.141785	8597319			
38	423.138370	42153784	$C_{20}H_{25}Na_2O_7^+$	423.1390	-1.46
37	423.135592	981815			
36	419.137205	595364			
35	416.175484	1012884			
34	415.235971	957944			
33	415.172136	3834998	$C_{21}H_{28}NaO_7^+$	415.1727	-1.50
32	414.232591	8750680			
31	413.229199	34502200	$C_{23}H_{34}NaO_5^+$	413.2298	-1.56
30	413.226460	759927			
29	406.002962	542501			
28	402.159829	1003135			
27	401.156484	4325459	$C_{20}H_{26}NaO_7^+$	401.1571	-1.43
26	397.225474	559391			
25	396.222062	4102512			
24	395.218719	15700801	$C_{23}H_{32}NaO_4^+$	395.2193	-1.47
23	386.201280	990881			
22	385.197999	3348853	$C_{21}H_{30}NaO_5^+$	385.1985	-1.41
21	378.211624	732243			
20	377.208180	2223918	$C_{23}H_{30}NaO_3^+$	377.2087	-1.37
19	367.187484	1777657	$C_{21}H_{28}NaO_4^+$	367.1880	-1.31
18	358.170102	4270660			
17	357.166725	19447348	$C_{19}H_{26}NaO_5^+$	357.1672	-1.52
16	357.164736	740660			
15	351.117398	1154918	$C_{17}H_{21}Na_2O_5^+$	351.1179	-1.39
14	349.176863	771538	$C_{21}H_{26}NaO_3^+$	349.1774	-1.48
13	322.434396	456130			
12	299.388673	441698			
11	288.128367	655280			
10	287.125005	3550047	$C_{15}H_{20}NaO_4$	287.1254	-1.32
9	287.124621	2741471			
8	277.578757	813656			
7	265.069528	1091029			
6	258.515795	437679			
5	255.060078	636610	$C_{11}H_{13}Na_2O_4^+$	255.0604	-1.07
4	251.125076	509646			
3	233.114493	1016911	$C_{12}H_{18}NaO_3^+$	233.1148	-1.35
2	231.098883	1478199	$C_{12}H_{16}NaO_3^+$	231.0992	-1.15

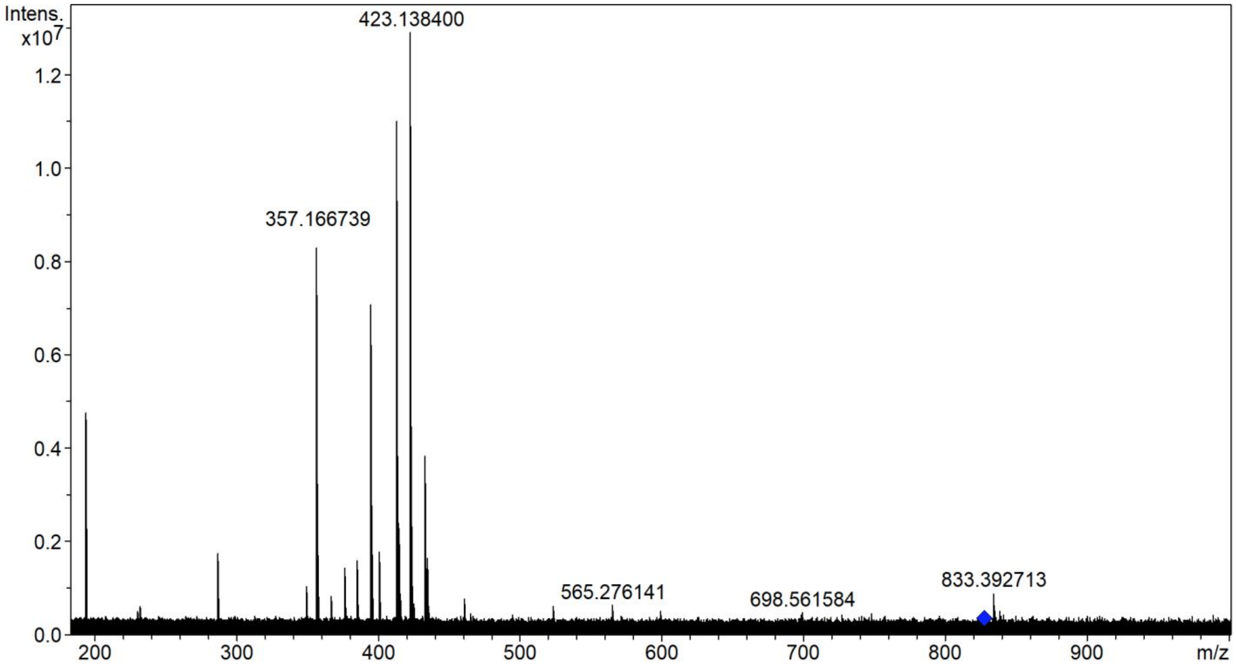

Figure S4. CID fragmentation of ^{18}O-GDA-sa, precursor ion *m/z* 833, Na^+ adduct. GDA-sa formed by sodium phosphate, pH 8.0 in 1:1 MeOH-$H_2{}^{18}O$. See Table 5.

#	Observed, *m/z*	Intensity	Formula	Calculated, *m/z*	Error, ppm
42	838.304673	516374			
41	834.396446	522126			
40	833.392713	910348	$C_{43}H_{61}Na_2O_{12}{}^{18}O^+$	833.3942	-2.10
39	698.603018	488152			
38	698.585600	490328			
37	698.561584	492119			
36	598.812955	503223			
35	598.779741	478840			
34	598.756369	467640			
33	565.276141	651082	$C_{31}H_{42}NaO_8{}^+$	565.2772	-1.93
32	523.944038	602329			
31	465.736431	473842			
30	460.764796	782729			
29	436.251577	573107			
28	435.248247	1647553	$C_{23}H_{36}NaO_5{}^{18}O^+$	435.2489	-1.61
27	434.247362	1195980			
26	434.235964	794060			
25	433.244044	3898069	$C_{23}H_{36}NaO_5{}^{18}O^+$	433.2447	-1.51
24	433.232630	2747093	$C_{23}H_{34}NaO_4{}^{18}O_2{}^+$	433.2332	-1.50
23	425.142718	680906	$C_{20}H_{25}Na_2O_6{}^{18}O^+$	425.1433	-1.32
22	424.141766	2746558			
21	423.138400	12951215	$C_{20}H_{25}Na_2O_7{}^+$	423.1390	-1.46
20	416.236901	864917			
19	416.175449	562963			
18	415.233491	2107640	$C_{23}H_{34}NaO_4{}^{18}O^+$	415.2341	-1.42
17	415.172102	2309110	$C_{21}H_{28}NaO_7{}^+$	415.1727	-1.50
16	414.232599	2901465			

15	413.229234	11222456	$C_{23}H_{34}NaO_5^+$	413.2298	-1.56
14	401.156528	1826777	$C_{20}H_{26}NaO_7^+$	401.1571	-1.43
13	396.222071	1971706			
12	395.218725	7109324	$C_{23}H_{32}NaO_4^+$	395.2193	-1.47
11	385.198007	1628171	$C_{21}H_{30}NaO_5^+$	385.1985	-1.41
10	377.208148	1428693	$C_{23}H_{30}NaO_3^+$	377.2087	-1.50
9	367.187532	856516	$C_{21}H_{28}NaO_4^+$	367.1880	-1.31
8	358.170090	1963576			
7	357.166739	8653357	$C_{19}H_{26}NaO_5^+$	357.1672	-1.52
6	350.388928	1053816			
5	287.125004	1631160	$C_{15}H_{20}NaO_4^+$	287.1254	-1.32
4	287.124597	1800632			
3	233.114475	607506	$C_{12}H_{18}NaO_3^+$	233.1148	-1.35
2	231.098881	518018	$C_{12}H_{16}NaO_3^+$	231.0992	-1.58

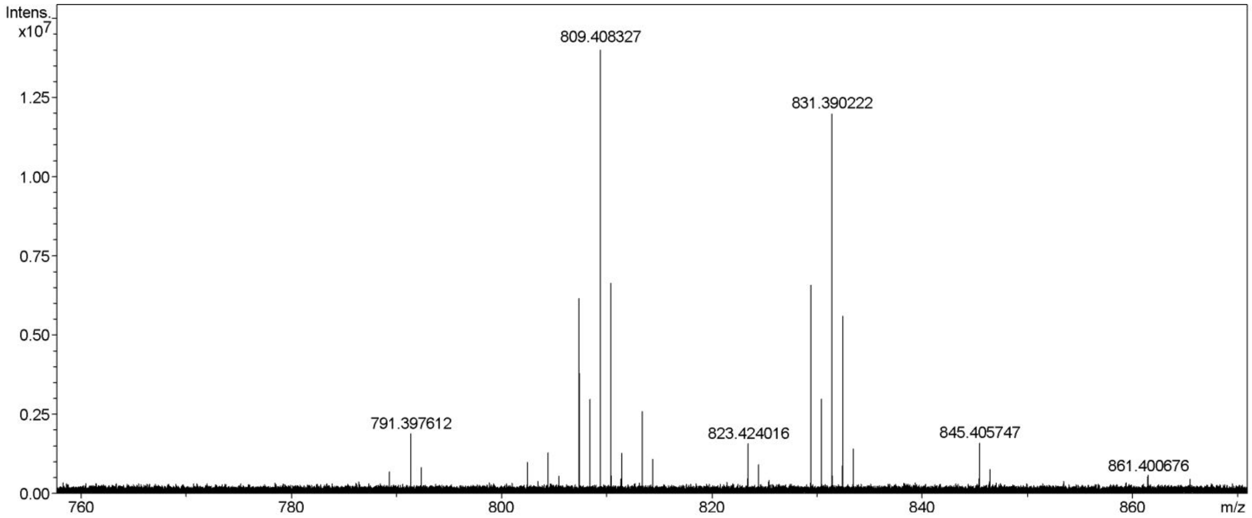

Figure S5. Reaction of GDA with anhydrous, methanolic Na_2CO_3, aqueous work-up, Na^+ adduct. See Table 7a.

#	Observed, *m/z*	Intensity	Formula	Calculated, *m/z*	Error, ppm
486	845.405747	1633554	$C_{44}H_{63}Na_2O_{13}^+$	845.4059	-0.13
483	831.390222	12012866	$C_{43}H_{61}Na_2O_{13}^+$	831.3902	0.02
480	829.374711	6612224	$C_{43}H_{59}Na_2O_{13}^+$	829.3746	0.19
478	823.424016	1590853	$C_{44}H_4NaO_{13}^+$	823.4239	0.13
476	813.379889	2586408	$C_{43}H_{59}Na_2O_{12}^+$	813.3796	0.30
473	809.408327	14097095	$C_{43}H_{62}NaO_{13}^+$	809.4083	0.08
471	807.392782	6257306	$C_{43}H_{60}NaO_{13}^+$	807.3926	0.21
470	805.456406	553329	$C_{38}H_{70}NaO_{16}^+$	85.4556	0.99
466	791.397612	1912230	$C_{43}H_{60}NaO_{12}^+$	791.3977	-0.11
465	789.382051	685974	$C_{43}H_{58}NaO_{12}^+$	789.3820	0.00
464	745.507257	523800	$C_{38}H_{74}NaO_{12}^+$	745.5072	0.01
462	695.303834	583844	$C_{36}H_{48}NaO_{12}^+$	695.3038	0.05
461	685.435554	1706627	$C_{43}H_{61}Na_2O_{13}^+$	831.3902	0.02

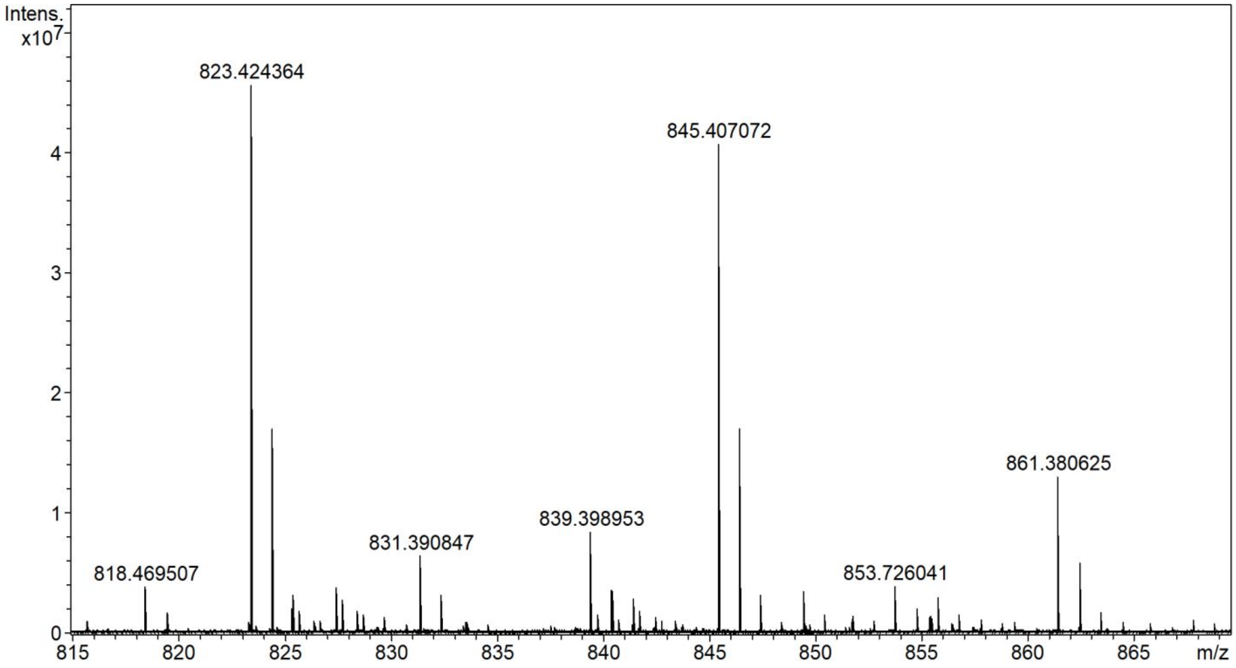

Figure S6. Polar product ions from reaction of GDA with anhydrous, methanolic ammonia, Na^+ adducts. See Table 7c.

Observed, *m/z*	Intensity	Formula	Calculated, *m/z*	ppm	Notes
845.4071	4.1e7	$C_{44}H_{63}Na_2O_{13}^+$	845.4059	1.47	GDA + MeOH - H^+ + $2Na^+$
823.4244	4.6e7	$C_{44}H_{64}NaO_{13}^+$	823.4239	0.59	GDA + MeOH + Na^+

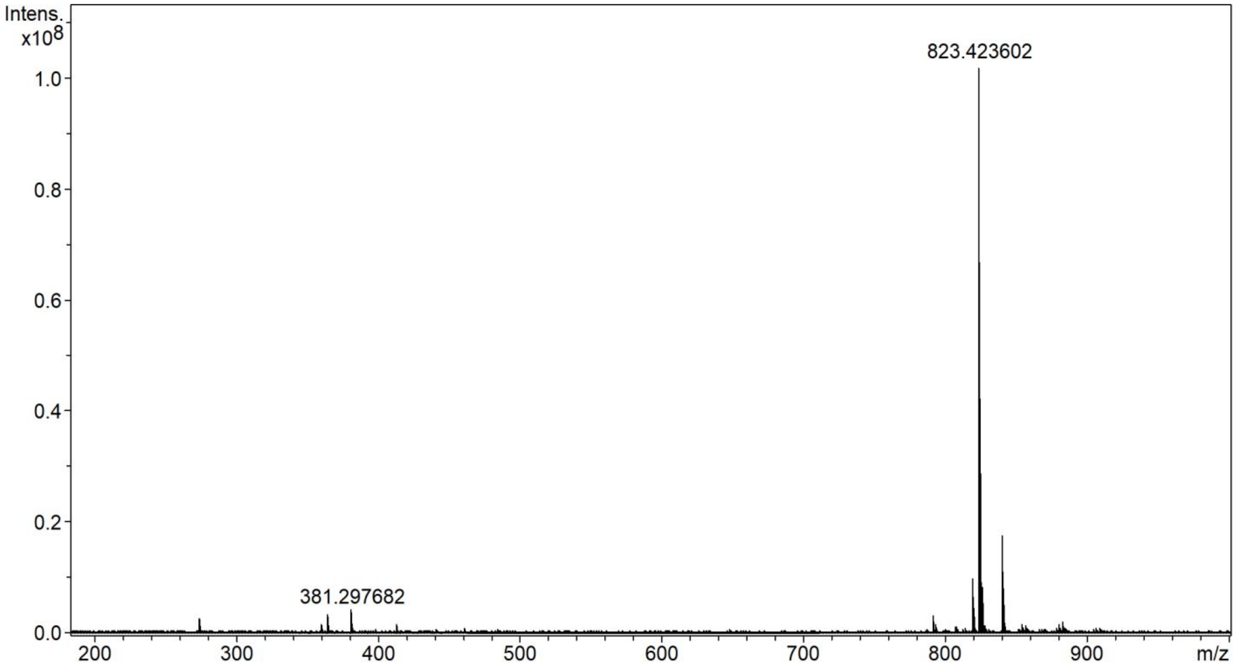

Figure S7. Methyl ester of GDA-sa, Na^+ adduct, *m/z* 823.4236, prepared with anhydrous, methanolic NH_3. See Table 7c.

#	Observed, *m/z*	Intensity	Formula	Calculated, *m/z*	Error, ppm
78	851.263897	568012			
75	841.725820	728581			
74	841.404698	1830398			
73	841.396028	1105918			
70	839.710318	714199			
69	839.398277	18289616			
65	829.726272	604778			
63	827.710611	1191100			
59	825.694505	822415			
58	825.430865	8254089			
57	825.427282	2119305			
53	823.433888	1017950			
52	823.432102	1578590			
51	823.430269	2169438			
50	823.428496	3961438			
49	823.426555	9192030			
48	823.423602	102740576	$C_{44}H_{64}NaO_{13}^+$	823.4239	-0.38
47	823.405071	896349			
45	819.472487	4369935			
43	813.694577	816046			
42	811.679081	598419			

40	807.412234	471912			
39	807.371886	1025384			
37	801.694527	415305			
35	799.678943	640196			
34	797.663536	419905			
33	793.404796	513473			
31	791.398287	3092803	$C_{43}H_{60}NaO_{12}^{+}$	791.3977	0.74

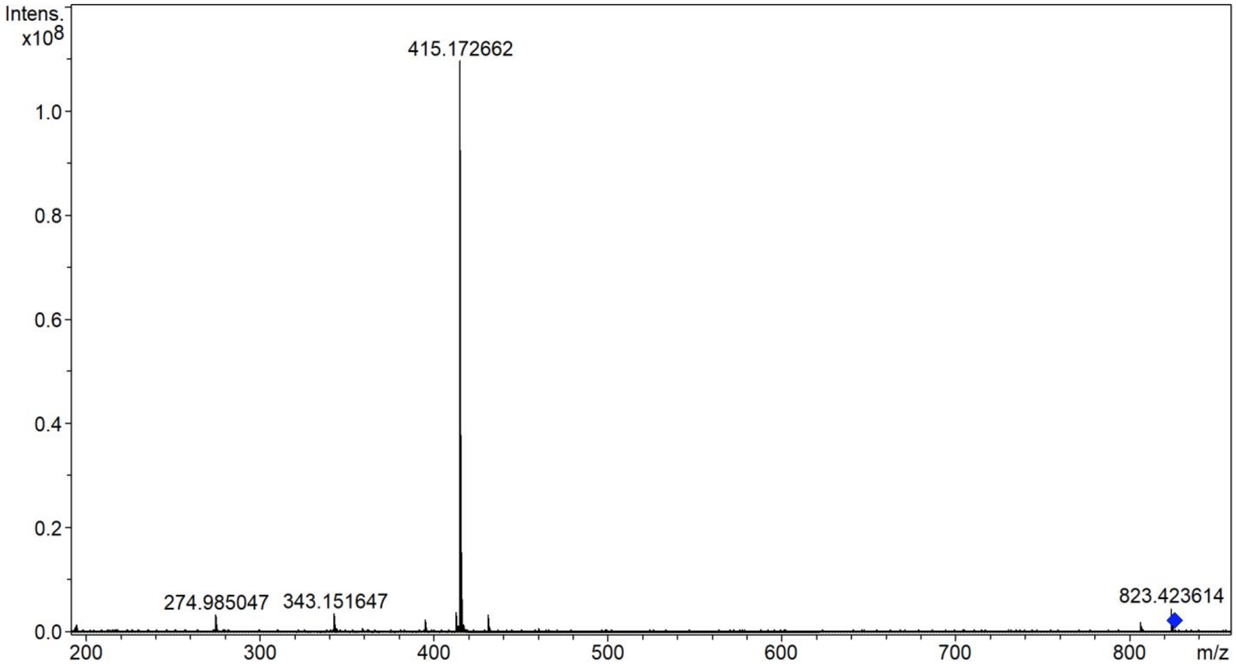

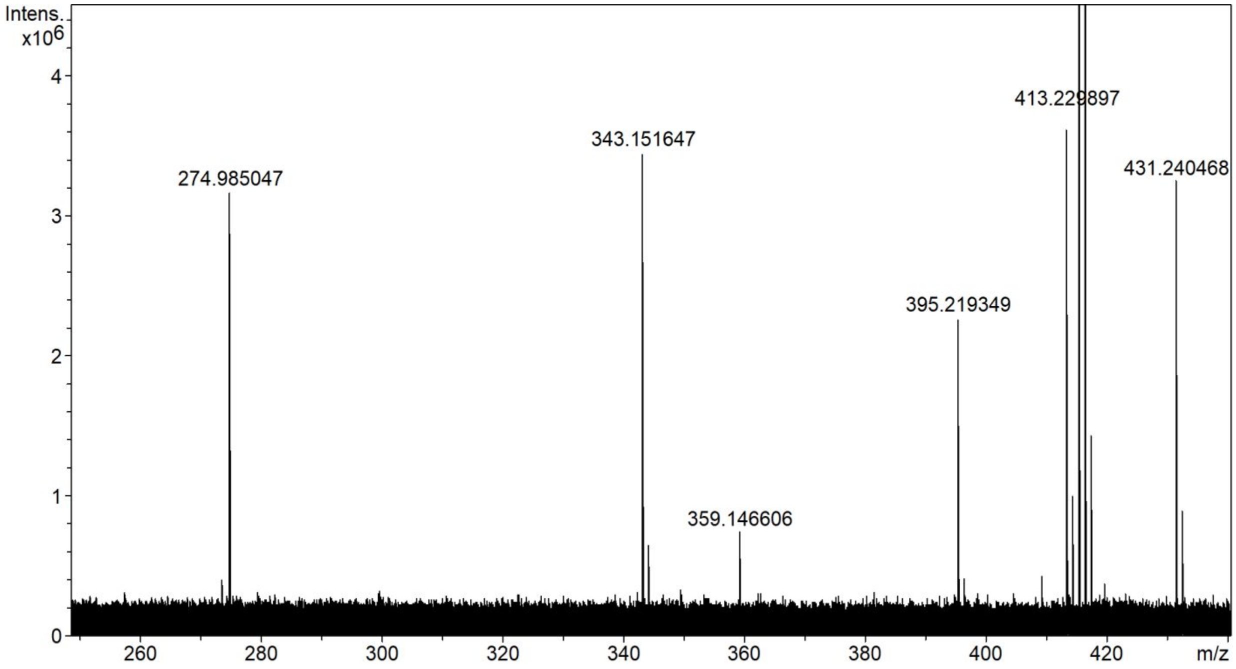

Figure S8. CID fragmentation of methanolysis product, *m/z* 823.4236, formed by reaction of GDA with anhydrous, methanolic NH_3. Na^+ adduct. See Table 7c.

#	Observed, *m/z*	Intensity	Formula	Calculate, *m/z*	Error
36	825.430769	439514			
34	823.423614	4479121	$C_{44}H_{64}NaO_{13}^+$	823.4239	-0.09
32	805.413519	2006201	$C_{44}H_{62}NaO_{12}^+$	805.4133	0.19

31	787.402711	415225	$C_{44}H_{60}NaO_{11}^{+}$	787.4028	-0.11
29	623.282566	493945	$C_{33}H_{44}NaO_{10}^{+}$	623.2827	-0.11
23	431.240468	3261928	$C_{23}H_{36}NaO_{6}$+	431.2404	0.21
22	419.466616	363810			
21	419.376411	374881			
20	417.179484	1446456			
19	417.177011	666040			
17	415.175326	1179408			
16	415.174405	2380048			
15	415.172662	109764880	$C_{21}H_{28}NaO_{7}^{+}$	415.1727	-0.06
14	415.169560	1493520			
12	413.229897	3757798	$C_{23}H_{34}NaO_{5}^{+}$	413.2298	0.13
11	409.153777	431047			
9	395.219349	2278934	$C_{23}H_{32}NaO_{4}^{+}$	395.2193	0.05
8	359.146606	750720	$C_{18}H_{24}NaO_{6}^{+}$	359.1465	0.25
6	343.151647	3466819	$C_{18}H_{24}NaO_{5}^{+}$	343.1516^{+}	0.02
4	273.673881	403600			
3	247.094193	348526			
